# Polystyrene nanoplastics impair mitochondrial function, quality control, and ER–mitochondria crosstalk in HeLa cells

**DOI:** 10.3389/ftox.2026.1907794

**Published:** 2026-10-05

**Authors:** Giuseppe Petito, Maria Teresa Gentile, Arianna Cuomo, Angelo Fusco, Sara Falvo, Maria della Valle, Gianluca D’Abrosca, Carla Isernia, Roberto Fattorusso, Maria Emanuela Errico, Antonia Lanni, Gaetano Malgieri, Rosalba Senese

**Affiliations:** 1 Department of Environmental, Biological, and Pharmaceutical Sciences and Technologies, University of Campania “Luigi Vanvitelli”, Caserta, Italy; 2 Institute of Crystallography, CNR, Caserta, Italy; 3 Department of Human Sciences, Link Campus University, Roma, Italy; 4 Institute for Polymers, Composites and Biomaterials, CNR, Pozzuoli, Italy

**Keywords:** HeLa cells, mitochondria associated membranes (MAMs), mitochondrial dysfunction, mitochondrial unfolded protein response (UPR^mt^), nanoplastics (NPs), oxidative stress

## Abstract

**Introduction:**

Polystyrene nanoplastics (PS-NPs) represent persistent environmental contaminants derived from the degradation of plastic debris and are increasingly detected in biological systems, raising concerns regarding their potential effects on human health. Emerging evidence suggests that, following cellular internalization, PS-NPs can induce intracellular stress responses and interfere with mitochondrial homeostasis, highlighting mitochondria as key targets of nanoplastic-induced cellular dysfunction. This study aimed to investigate the effects of PS-NPs on mitochondrial function in HeLa cells. Particular attention was devoted to Mitochondrial Quality Control (MQC) pathways, including the mitochondrial Unfolded Protein Response (UPR^mt^), a stress-adaptive mechanism that remains poorly characterized in response to nanoplastic exposure in human cellular model. In addition, the study examined the functional crosstalk between the Endoplasmic Reticulum and mitochondria.

**Methods:**

HeLa cells were exposed to PS-NPs, and mitochondrial bioenergetics, oxidative stress, mitochondrial membrane potential, cellular ATP levels, extracellular lactate levels, and calcium homeostasis were assessed using functional assays. MQC-related pathways, including UPR^mt^, mitochondrial biogenesis, dynamics, autophagy/mitophagy, and ER stress responses, were evaluated through biochemical, molecular, and imaging approaches.

**Results:**

Our results demonstrate that PS-NP exposure induces mitochondrial dysfunction, characterized by impaired mitochondrial coupling, mitochondrial membrane depolarization, increased H_2_O_2_ levels in cell lysates, and altered cellular redox homeostasis. Moreover, PS-NPs disrupt MQC processes, leading to an imbalance in the mechanisms that maintain mitochondrial homeostasis, including modulation of UPR^mt^-associated proteins and dysregulation of ER–mitochondria communication via Mitochondria-Associated Membranes (MAMs).

**Conclusion:**

Collectively, these data advance our understanding of PS-NP toxicity by identifying alterations in MQC systems and ER–mitochondria crosstalk as key events in nanoplastic-induced cellular stress. These findings provide novel mechanistic insights into nanoplastic toxicity and highlight the potential impact of environmental PS-NP exposure on cellular homeostasis and human health.

## Graphical Abstract

**Figure:**
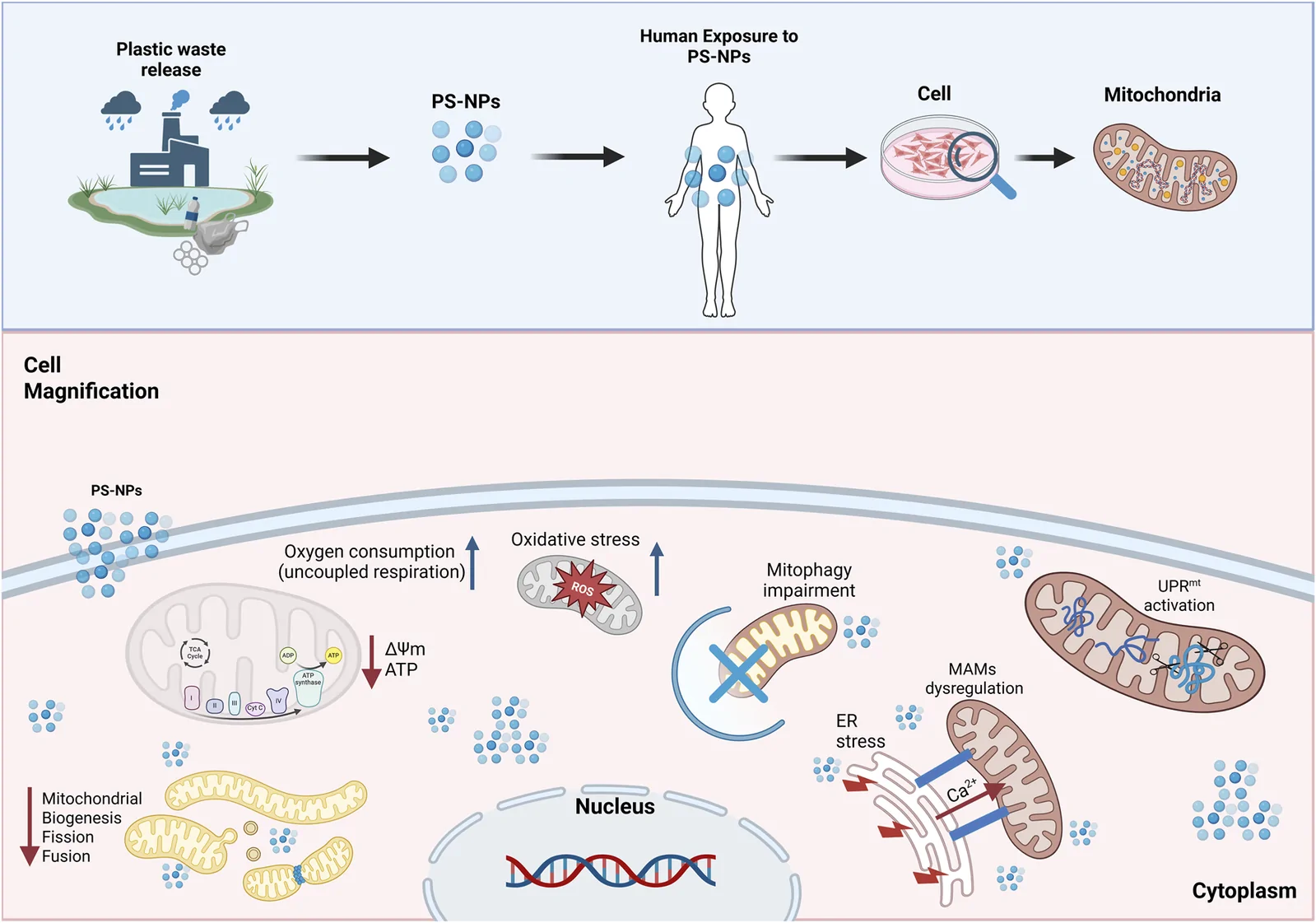

## 1 Introduction

The extensive use of plastic-based materials has led to increasing concerns about their potential negative effects on human health. These plastics are classified by size as micro and nanoplastics (Rubio et al., 2020). Microplastics (MPs) are plastic fragments smaller than 5 mm, generated either through the breakdown of larger plastic debris or intentionally produced for use in commercial applications (Zhang et al., 2021). Nanoplastics (NPs) are typically defined as particles smaller than 1 μm. They represent a potential environmental threat because they can form as temporary by-products during microplastic degradation. In addition, they may pose risks at high concentrations, even when these are difficult to detect (Jiang et al., 2020). People are continuously exposed to MPs and NPs through inhalation of contaminated air, ingestion of polluted food and water, and dermal contact (Xu et al., 2022). NPs can readily translocate from environmental compartments into living organisms, including humans, highlighting their environmental and health relevance as emerging contaminants with relevance to ecosystem integrity and human exposure (Thakur et al., 2025).

The presence of nanoparticles in the human body can elicit cellular stress and inflammatory responses, which may contribute to tissue injury and impair organ function, highlighting potential risks to human health (Xuan et al., 2023). Studies have demonstrated that, following oral exposure, polystyrene nanoparticles (PS-NPs) can reach multiple tissues and organs and be systemically distributed throughout the body (Xu et al., 2021; Deng et al., 2017). Moreover, other studies have confirmed that chronic exposure to PS-NPs can lead to ventricular dilation, reduced myocardial contractility and decreased blood pressure in mice (Xiong et al., 2025). Evidence from *in vivo* and *in vitro* studies suggests that PS-NP exposure promotes hepatic oxidative stress by enhancing reactive oxygen species levels and downregulating the Nrf2 antioxidant pathway (Wen et al., 2024). These effects are consistent with a broader pattern of oxidative stress–mediated and mitochondria-related toxicity observed across different organ systems.

Upon cellular internalization, PS-NPs may associate with intracellular components and thereby perturb physiological cellular functions (Bang et al., 2025; Bu et al., 2024; Li et al., 2023; Milillo et al., 2024). Due to their small size and chemical characteristics, NPs can interact directly with intracellular components, including mitochondria (Li et al., 2023; Huang et al., 2023; Wang et al., 2021). Mitochondria are central regulators of cellular metabolism, responsible for ATP production through oxidative phosphorylation and for the control of calcium homeostasis, apoptosis, and reactive oxygen species generation. In SH-SY5Y human neuroblastoma cells, PS-NP exposure leads to mitochondrial dysfunction (Tang et al., 2022; Fan et al., 2024). Moreover, Huang et al. demonstrated that 20 nm PS-NPs are efficiently taken up by macrophages and localize to mitochondria, leading to reduced ATP synthesis and activation of apoptotic signaling pathways, which ultimately results in cellular toxicity (Huang et al., 2023).

Notably, the cellular response to PS-NPs is highly dependent on nanoparticle size, concentration, and length of exposure. However, although some studies have investigated the genotoxic effects of PS-NPs in HeLa cells (Feola et al., 2025), comprehensive evidence regarding their impact on the mitochondrial compartment and its functional aspects is still lacking.

In this context, the present study aims to evaluate the impact of PS-NPs on mitochondrial function in HeLa cells, with a specific focus on the Mitochondrial Quality Control (MQC) system, a dynamic network essential for maintaining mitochondrial integrity under stress conditions, thereby providing a comprehensive overview of how nanoplastics may disrupt mitochondrial compartment integrity. Additionally, we examined the effects of PS-NPs exposure on the interaction between the Endoplasmic Reticulum and mitochondria, with particular emphasis on MAMs, which are essential for Ca^2+^ homeostasis, redox signaling and the regulation of apoptosis. Furthermore, we investigated the involvement of mitochondrial Unfolded Protein Response (UPR^mt^)-associated mechanisms. To the best of our knowledge, this study provides the first evidence that PS-NP exposure induces a mitochondrial stress response associated with the upregulation of key UPR^mt^-related proteins in human cells.

Collectively, this study provides new insights into the mechanisms by which PS-NPs may impair mitochondrial homeostasis and compromise cellular functions, highlighting mitochondrial dysfunction as a potential link between environmental nanoplastic exposure and adverse effects on human health.

## 2 Materials and methods

### 2.1 PS-NPs characterization

Polystyrene nanoplastics (PS-NPs, 1% solids) were obtained from Thermo Fisher Scientific–Particle Technology (CA, United States; Catalog #3020A, lot #273137). According to the manufacturer’s specifications (www.thermofisher.com), the particles exhibited an average diameter of 22 ± 2 nm, a density of 1.05 g cm^-3^ at ambient temperature, and a refractive index of 1.59 at 589 nm. The nanoparticles employed in this study were selected because they represent a well-characterized experimental model widely used in mechanistic nanotoxicology studies. In particular, PS-NPs within the ∼20–25 nm size range have been extensively applied to investigate cellular uptake, intracellular trafficking, and toxicity mechanisms (Ruan et al., 2023; Adamiak et al., 2025). Importantly, this nanoparticle formulation has also been previously employed in our previous studies in HeLa cells, where it was shown to induce reproducible genotoxic and cytotoxic effects as well as to interact with intracellular proteins, including ubiquitin, affecting cellular protein homeostasis (Feola et al., 2025; Della Valle et al., 2022). To minimize aggregation and ensure uniform dispersion, the PS-NPs were subjected to sonication at 25% amplitude for 10 min prior to each experiment using a Sonics Vibracell ultrasonic processor (500 W, 20 kHz; Newtown, CT, United States). PS-NPs were also characterized in the cell medium in order to confirm their specifications and behavior in the conditions chosen for the following experiments. 2 μg/mL dispersion in Roswell Park Memorial Institute (RPMI-1640) medium was investigated by Dynamic Light Scattering (DLS) and Electrophoretic Light Scattering (ELS) using a Malvern Panalytical (Malvern, UK) Zetasizer Pro. All measurements were performed four times at the temperature of 25 °C, confirming an average particle size of approximately 23 nm and resulting in a zeta potential of −45 mV.

### 2.2 Cell culture and treatment

HeLa 229 cells (ATCC, Rockville, MD, United States) were maintained in Roswell Park Memorial Institute (RPMI-1640) medium under standard culture conditions (Thermo Fisher Scientific, Waltham, MA, United States). The growth medium was supplemented with 10% Fetal Bovine Serum, 1% penicillin–streptomycin, and 200 mM L-glutamine (Thermo Fisher Scientific, Waltham, MA, United States). Cells were incubated at 37 °C in a humidified atmosphere containing 5% CO_2_. For experimental treatments, cells were exposed to 2 μg/mL PS-NPs dispersed in RPMI-1640 for either six or 24 h at 37 °C. We chose this specific concentration to reflect actual biological exposure scenarios, according to recent data (Kadac-Czapska et al., 2024). While environmental monitoring generally detects lower levels of nanoplastics in food and water, ingested particles may resist efficient clearance, leading to progressive tissue accumulation (Vitali et al., 2023). By using higher concentration, we simulated both immediate acute exposure and the elevated intracellular burdens associated with long-term increase, while remaining within a concentration range comparable to that found in human blood (Leslie et al., 2022). At last, cells were collected by gentle scraping, washed twice with phosphate-buffered saline (PBS), and stored at −80 °C for subsequent analyses.

### 2.3 MTT assay

HeLa cells were plated in 96-well plates at a density of 1.5 × 10^4^ cells per well in 100 μL of complete medium, with each condition performed in triplicate. After 24 h, cells were exposed to PS-NPs at concentrations of 0.2, 2, and 20 μg/mL for six or 24 h. Subsequently, 150 μL of MTT solution (Merck, Darmstadt, Germany), prepared in serum-free medium, was added to each well and incubated for 4 h at 37 °C in a humidified atmosphere containing 5% CO_2_. After incubation, the MTT solution was removed, and the resulting formazan crystals were solubilized with 100 μL of dimethyl sulfoxide (DMSO) (Merck, Darmstadt, Germany). Absorbance was measured at 570 nm using a Synergy H1 microplate reader (BioTek, Agilent Technologies, Santa Clara, CA, United States).

### 2.4 Trypan Blue exclusion assay

Cell viability was also assessed using the Trypan Blue exclusion assay. HeLa cells were seeded in 24-well plates and exposed to PS-NPs at concentrations of 0.2, 2, and 20 μg/mL for six or 24 h. Following treatment, cells were harvested by trypsinization, resuspended in complete medium, and mixed 1:1 with 0.4% Trypan Blue solution (Thermo Fisher Scientific, Waltham, MA, United States). Viable (unstained) and non-viable (blue-stained) cells were counted using a Countess II Automated Cell Counter (Thermo Fisher Scientific, Waltham, MA, United States). Cell viability was expressed as the percentage of viable cells relative to the total cell population and was calculated as follows: viable cells/(viable + non-viable cells) × 100.

### 2.5 Evaluation of oxygen consumption rate (OCR) of intact cells

Oxygen consumption rate (OCR) was measured in intact HeLa cells using an Oxygraph-2k high-resolution respirometer (Oroboros Instruments, Innsbruck, Austria). Following trypsinization, cells were collected in RPMI-1640 medium and centrifuged at 350 *g* for 5 min at room temperature. The resulting cell pellet was resuspended in RPMI-1640 medium at a density of 1 × 10^6^ cells/mL. After instrument calibration, 2 mL of the cell suspension was transferred into each respirometer chamber. Basal respiration was recorded before the addition of mitochondrial inhibitors. Oligomycin (2.5 µM; Merck, Darmstadt, Germany) was subsequently added to inhibit ATP synthase, allowing measurement of post-oligomycin OCR. FCCP (carbonyl cyanide-4-(trifluoromethoxy)phenylhydrazone; 1 μM; Merck) was then added to stimulate maximal respiratory activity. Finally, rotenone (0.5 µM; Merck) and antimycin A (2.5 µM; Merck) were added to inhibit mitochondrial complexes I and III, respectively, thereby allowing the measurement of residual non-mitochondrial oxygen consumption. Steady-state OCR values were extracted from each respiratory phase by applying the same selection criterion to all biological replicates and experimental groups.

ATP-linked respiration and proton-leak respiration were calculated according to Pesta and Gnaiger (Pesta and Gnaiger, 2012), after correction for residual oxygen consumption measured following rotenone/antimycin A addition, using the following equations:

$$\text{ATP}-\text{linked respiration}=\left(\text{basal OCR }-\text{Rot}/\text{AA OCR}\right)-\left(\text{post}-\text{oligomycin OCR }-\text{Rot}/\text{AA OCR}\right)$$

Proton−leak respiration=post−oligomycin OCR−Rot/AA OCR

Because the residual OCR term is subtracted from both respiratory states, the ATP-linked respiration equation is mathematically equivalent to basal OCR − post-oligomycin OCR.

All respiratory parameters were calculated separately for each biological replicate, and OCR values were normalized to 10^6^ cells. Detailed experimental information is provided in the Supplementary Section S1.

### 2.6 Assessment of cellular ATP levels

Cellular ATP levels were quantified using the Adenosine 5′-triphosphate (ATP) Assay Kit (Abbexa, Cambridge Science Park, UK) following the manufacturer’s instructions. ATP levels were measured at 636 nm using a Synergy H1 microplate reader (BioTek, Shoreline, WA, United States) and normalized to total protein. Detailed experimental information is provided in the Supplementary Section S2.

### 2.7 Measurement of extracellular lactate levels

Extracellular L-lactate levels were quantified using a colorimetric L-Lactic Acid Assay Kit (Elabscience Biotechnology, Wuhan, China; catalog no. E-BC-K044-M). HeLa cells were left untreated or exposed to 2 μg/mL PS-NPs for six or 24 h under the experimental conditions described above. At the end of each exposure period, aliquots of conditioned culture medium were collected and processed according to the manufacturer’s protocol. Samples, blanks and L-lactate standards were analyzed in parallel, and absorbance was measured at 530 nm using a Synergy H1 microplate reader (BioTek, Agilent Technologies, Santa Clara, CA, United States). L-lactate concentrations were determined from the corresponding standard curve.

### 2.8 Mitochondrial membrane potential assay (MMP)

MMP was evaluated using the TMRE Mitochondrial Membrane Potential Assay Kit (Catalog no. ab113852, Abcam, UK), following previously described protocols (Petito et al., 2025a). TMRE is a positively charged, cell-permeable fluorescent dye that accumulates in polarized mitochondria in a membrane-potential-dependent manner; therefore, a reduction in TMRE fluorescence indicates mitochondrial depolarization. TMRE fluorescence in mitochondria was visualized with a Leica DMLB fluorescence microscope (Leica, Wetzlar, Germany).

### 2.9 MitoBright green staining

MitoBright Green staining was performed using the MitoBright Green Probe Assay Kit (catalog no. E-CK-A401, Elabscience Biotechnology, Wuhan, China) according to the manufacturer’s protocol. According to the manufacturer, MitoBright Green E-CK-A401 is a cell-permeable fluorescent probe that labels intact mitochondria in living cells by binding to negatively charged mitochondrial membrane phospholipids, particularly cardiolipin, and can be used for the qualitative or quantitative assessment of mitochondrial mass. Briefly, HeLa cells were seeded and exposed to PS-NPs under the experimental conditions described above. Following treatment, cells were incubated with MitoBright Green staining solution for 30 min at 37 °C in the dark. Cells were subsequently washed with PBS to remove excess probe, and fluorescence images were immediately acquired using a Leica DMLB fluorescence microscope (Leica Microsystems, Wetzlar, Germany).

### 2.10 hydrogen peroxide measurement in cell lysates

Hydrogen peroxide (H_2_O_2_) levels were measured using a commercial fluorometric assay kit (Abcam, ab102500). Following PS-NP exposure, the culture medium was completely removed and the cells were washed to minimize any contribution from extracellular H_2_O_2_. A total of 2 × 10^6^ cells were collected, resuspended in Assay Buffer and lysed according to the manufacturer’s instructions. The cell lysates were centrifuged at 14,000 × g for 2–5 min at 4 °C, and the resulting supernatants were collected. To remove proteins, the lysate supernatants were treated with 4 M perchloric acid (PCA) and subsequently neutralized with ice-cold 2 M KOH to a final pH of 6.5–8.0. The neutralized samples were centrifuged at 13,000 × g for 15 min at 4 °C, and the resulting clarified supernatants were used for H_2_O_2_ quantification. According to the manufacturer’s instructions, 50 µL of each sample was mixed with 50 µL of the reaction mixture in a 96-well plate. Following incubation, fluorescence was measured at 535 nm excitation and 587 nm emission using a Synergy H1 microplate reader (BioTek, Agilent, Santa Clara, CA, United States). Detailed experimental information is provided in the Supplementary Section S3.

### 2.11 Western blotting

Cells were rinsed with PBS and lysed on ice for 30 min in RIPA buffer supplemented with protease and phosphatase inhibitors, according to a previously reported method (Petito et al., 2025a; Petito et al., 2025b). Protein extracts were separated by SDS-PAGE and transferred onto Nitrocellulose membranes (Billerica, MA, United States). Membranes were blocked with 5% nonfat dry milk in TBS-T. Primary antibodies were diluted in TBS-T with 5% BSA, and secondary antibodies in TBS-T with 5% nonfat dry milk. Horseradish peroxidase-conjugated secondary antibodies allowed chemiluminescent detection using the Chemi-Doc system (Bio-Rad, Hercules, CA, United States), and protein bands were quantified with ImageLab 6.1 (Bio-Rad). A full list of antibodies is provided in Supplementary Table S1.

### 2.12 Genomic DNA isolation and mtDNA copy number by Real-Time PCR

Genomic DNA was extracted from HeLa cells using the Genomic-tip 20/G kit (Qiagen, Valencia, CA, United States) according to the manufacturer’s protocol, and quantified with a Nanodrop One spectrophotometer (Thermo Fisher Scientific, Waltham, MA, United States). mtDNA copy number was determined by qRT-PCR using the StepOnePlus™ Real-Time PCR System (Thermo Fisher Scientific, Waltham, MA, United States), targeting the mitochondrial Cytochrome c oxidase subunit II (COII) and the nuclear β-actin gene. Detailed experimental information and primer sequences are provided in the Supplementary Section S4; Supplementary Table S2.

### 2.13 Autophagy detection

Autophagy was evaluated using the Autophagy Assay Kit (Sigma-Aldrich, Saint Louis, MO, United States) according to the manufacturer’s instructions. Cells (1 × 10^4^ per well) were plated in 12-well plates and, after PS-NP exposure, incubated with 100 µL of Autophagosome Detection Reagent for 30 min. Subsequently, fluorescence was detected using a Leica DMLB microscope (Leica, Wetzlar, Germany), and autophagic puncta were quantified per cell using ImageJ.

### 2.14 Intracellular Ca^2+^ determination

Intracellular Ca^2+^ levels were determined using a Calcium Assay Kit (Abbexa, Cambridge Science Park, UK). Absorbance was measured at 610 nm using a Synergy H1 microplate reader (BioTek, Agilent, Santa Clara, CA, United States). Data were normalized to total protein content.

### 2.15 Statistical analysis

Data were analyzed using GraphPad Prism 8.0.1 (GraphPad Software, San Diego, CA, United States). Data are presented as individual data points with mean ± standard deviation (SD). All experiments were performed using five independent biological replicates. For normally distributed data, statistical comparisons were performed using one-way ANOVA followed by Tukey’s *post hoc* test, or Student’s t-test for pairwise comparisons. A p-value <0.05 was considered statistically significant.

## 3 Results

### 3.1 PS-NP exposure alters mitochondrial bioenergetics and cellular energy metabolism in HeLa cells

To assess the impact of PS-NPs on cellular homeostasis, HeLa cell viability was first evaluated after exposure to 0.2, 2, and 20 μg/mL PS-NPs for 6 and 24 h using the MTT assay. Cell viability decreased in both a dose- and time-dependent manner upon PS-NP exposure. After 6 h, viability was comparable to controls at 0.2 μg/mL, decreased to 80%–90% at 2 μg/mL, and dropped to approximately 15% at 20 μg/mL (Figure 1A). After 24 h, viability ranged from 95% to 100% at 0.2 μg/mL, ∼80% at 2 μg/mL, and declined drastically to ∼10% at 20 μg/mL (Figure 1B). Consistent with the MTT assay, the Trypan Blue exclusion assay revealed a similar dose- and time-dependent reduction in cell viability following PS-NP exposure (Figures 1C,D). Based on these results, 2 μg/mL PS-NPs was selected for subsequent mechanistic investigations, as this concentration induced measurable cellular alterations while maintaining sufficient cell viability for downstream analyses.

**FIGURE 1:**
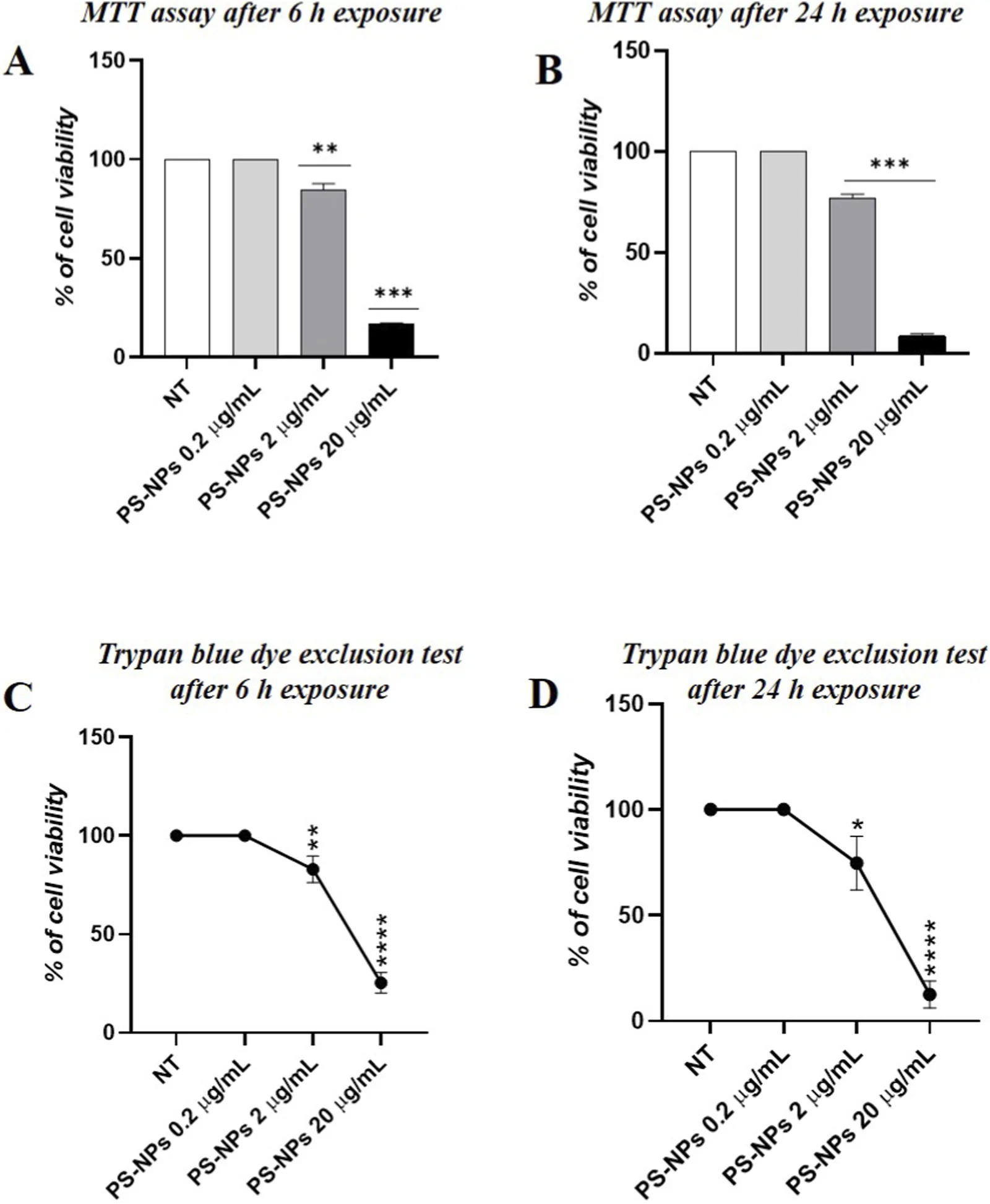
*HeLa Cell Viability After PS-NPs Exposure*. **(A,B)** HeLa cell viability after 6 and 24 h exposure to 0.2, 2, and 20 μg/mL PS-NPs, assessed by MTT assay. (**C,D**) Cell viability assessed by the Trypan Blue exclusion assay after exposure to 0.2, 2, and 20 μg/mL PS-NPs for 6 and 24 h. Data are shown as individual data points with mean ± SD from n = 5 independent biological replicates. Statistical significance was determined by one-way ANOVA with Dunnett’s *post hoc* test (*p < 0.05; **p < 0.01, ***p < 0.001, ****p < 0.0001 vs. NT).

To evaluate the effects of PS-NP exposure on mitochondrial function, OCR was measured in intact HeLa cells using high-resolution respirometry. Basal OCR was significantly increased after both 6 and 24 h of PS-NP exposure compared with untreated cells (Figure 2A). Post-oligomycin OCR showed an approximately 30% increase after 6 h and increased further after 24 h, when the difference reached statistical significance compared with untreated cells (Figure 2A). Similarly, FCCP-stimulated OCR was significantly increased at both exposure times, whereas rotenone/antimycin A-insensitive OCR remained unchanged (Figure 2A). To further characterize these bioenergetic alterations, ATP-linked respiration, proton-leak respiration and coupling efficiency were calculated separately for each biological replicate. ATP-linked respiration remained unchanged following PS-NP exposure (Figure 2B). In contrast, proton-leak respiration was significantly increased after both 6 and 24 h of exposure (Figure 2C). Coupling efficiency showed a progressive reduction, decreasing by approximately 15% relative to untreated cells after 6 h and reaching a statistically significant reduction after 24 h (Figure 2D). In parallel, cellular ATP levels were significantly reduced after both 6 and 24 h of PS-NP exposure compared with untreated cells (Figure 2E). Extracellular L-lactate concentrations were significantly increased after both exposure times compared with untreated cells (Figure 2F). TMRE fluorescence intensity was also significantly decreased at both exposure times, indicating mitochondrial membrane depolarization (Figures 2G,H). Collectively, the unchanged ATP-linked respiration, increased proton-leak respiration and reduced coupling efficiency indicate an alteration of mitochondrial bioenergetics characterized by impaired mitochondrial coupling following PS-NP exposure.

**FIGURE 2:**
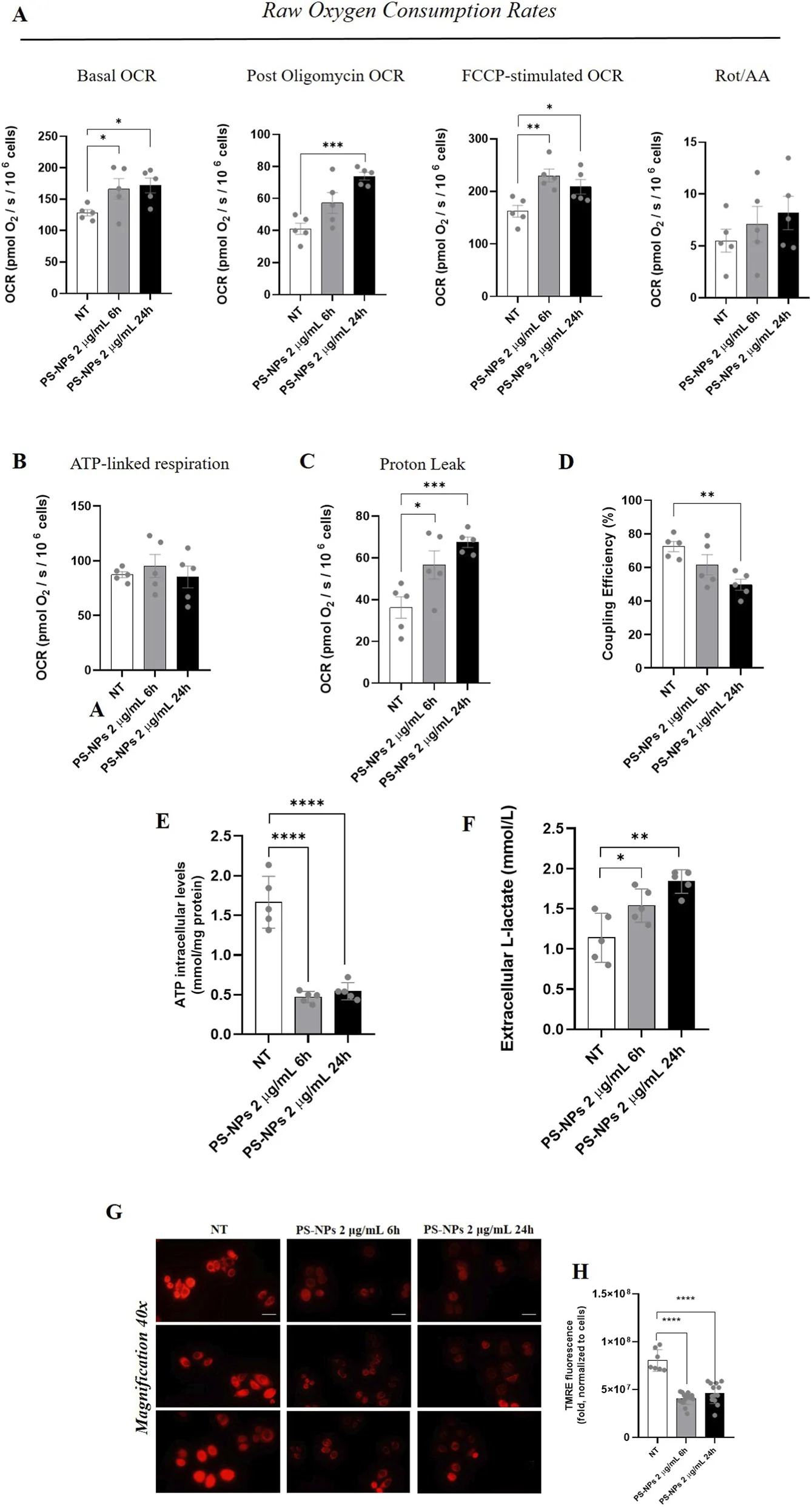
*PS-NP exposure alters mitochondrial bioenergetics and cellular energy metabolism in HeLa cells.* **(A)** Basal OCR, post-oligomycin OCR, FCCP-stimulated OCR and rotenone/antimycin A-insensitive OCR measured by high-resolution respirometry using an Oroboros O2k instrument. Oxygen consumption rates were normalized to 10^6^ cells. **(B–D)** Respiratory parameters calculated separately for each biological replicate: ATP-linked respiration **(B)**; proton-leak respiration **(C)**; and coupling efficiency **(D)**. **(E)** Cellular ATP levels following PS-NP exposure, expressed as mmol/mg protein. **(F)** Extracellular L-lactate concentrations measured in conditioned culture medium collected from untreated and PS-NP-exposed cells and expressed as mmol/L. **(G)** Representative fluorescence microscopy images of TMRE staining in untreated and PS-NP-exposed cells. Images were acquired at ×40 magnification using identical acquisition settings across all experimental groups. Scale bars: 40 µm. **(H)** Quantification of TMRE fluorescence intensity normalized to the number of analyzed cells. Data are presented as individual data points with mean ± SD from n = 5 independent biological replicates. Statistical significance was assessed using one-way ANOVA followed by Tukey’s multiple-comparisons test. *p < 0.05, **p < 0.01, ***p < 0.001, ****p < 0.0001 vs. NT.

### 3.2 PS-NP exposure alters redox homeostasis in HeLa cells

H_2_O_2_ levels measured in HeLa cell lysates were significantly increased following PS-NP exposure for 6 and 24 h compared with untreated controls (Figure 3A). To further characterize the cellular response to PS-NP-induced oxidative stress, we analyzed changes in the protein levels of key antioxidant defense enzymes. Specifically, we focused on catalase, SOD-2, glutathione peroxidase 1 (GPX-1), and glutathione peroxidase 4 (GPX-4), which play critical roles in ROS detoxification and the maintenance of cellular redox homeostasis (Jomova et al., 2024). Based on these observations and considering the time required for transcriptional and translational responses, subsequent analyses were performed after 24 h of PS-NP exposure. Our results revealed a significant decrease in SOD-2, GPX-1, and GPX-4 protein levels in cells exposed to PS-NPs for 24 h, whereas catalase levels remained unchanged compared with the control condition (Figure 3B). Together, the increase in cell-lysate H_2_O_2_ levels and the reduction in key antioxidant defense enzymes indicate that PS-NP exposure alters redox homeostasis and promotes an oxidative-stress-associated response in HeLa cells.

**FIGURE 3:**
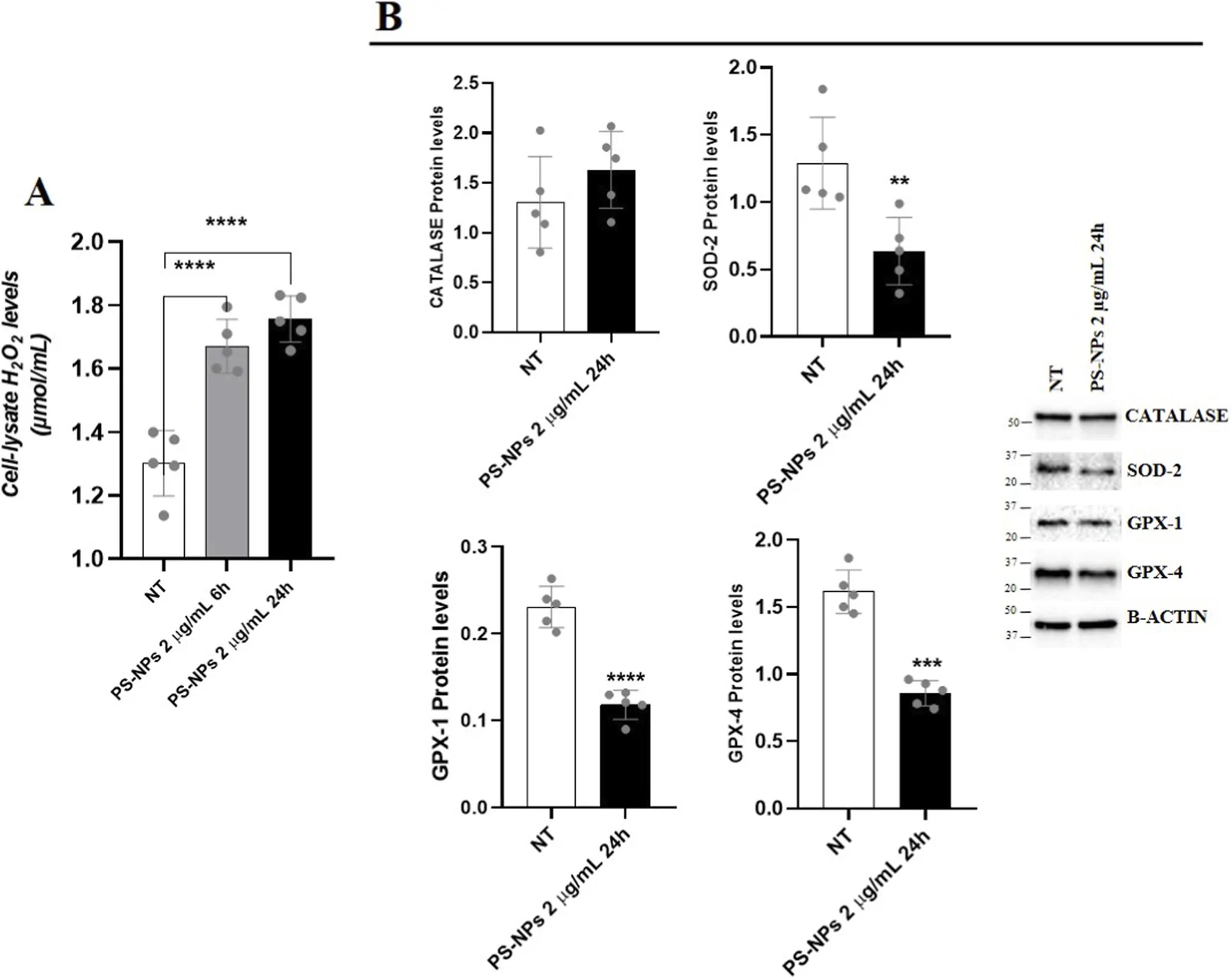
*PS-NP exposure alters redox homeostasis in HeLa cells.* **(A)** H_2_O_2_ levels measured in the clarified supernatants obtained from lysates of HeLa cells exposed to 2 μg/mL PS-NPs for six or 24 h. Results are expressed as µmol/mL of cell lysate. **(B)** Representative immunoblots showing CATALASE, SOD-2, GPX-1, and GPX-4 in cells exposed to 2 μg/mL PS-NPs for 24 h. Protein levels were normalized to β-ACTIN, and densitometric quantification is shown. The displayed blot is a representative cropped image; full-length uncropped blots are provided in the Supplementary Material. Data are shown as individual data points with mean ± SD from n = 5 independent biological replicates. Statistical analysis was determined using one-way ANOVA with Tukey’s *post hoc* test or Student’s t-test where appropriate. **p < 0.01, ***p < 0.001, ****p < 0.0001 vs. NT.

### 3.3 PS-NPs exposure alters MQC mechanisms in HeLa cells

Oxidative stress and mitochondrial dysfunction may be closely interconnected. To this aim, we investigated most of the processes involved in the MQC, including the UPR^mt^, mitochondrial biogenesis, fission, fusion, and autophagy/mitophagy. The canonical UPR^mt^ axis was analyzed in HeLa cells exposed to PS-NPs for 24 h. After 24 h of exposure, cells showed a significant increase in mitochondrial proteases, including CLPP (caseinolytic mitochondrial matrix peptidase proteolytic subunit) and LONP1 (Lon peptidase 1) protein levels, compared to controls (Figure 4A). Furthermore, the mitochondrial chaperone TRAP1 (tumor necrosis factor receptor-associated protein 1) and the transcription factor CHOP protein levels were markedly upregulated following PS-NP treatment (Figure 4A). Importantly, ATF5 (activating transcription factor 5) protein levels increased by approximately 18% in treated cells compared to controls (Figure 4A). These results suggest the involvement of UPR^mt^-associated mechanisms in the cellular response to PS-NP-induced mitochondrial stress.

**FIGURE 4:**
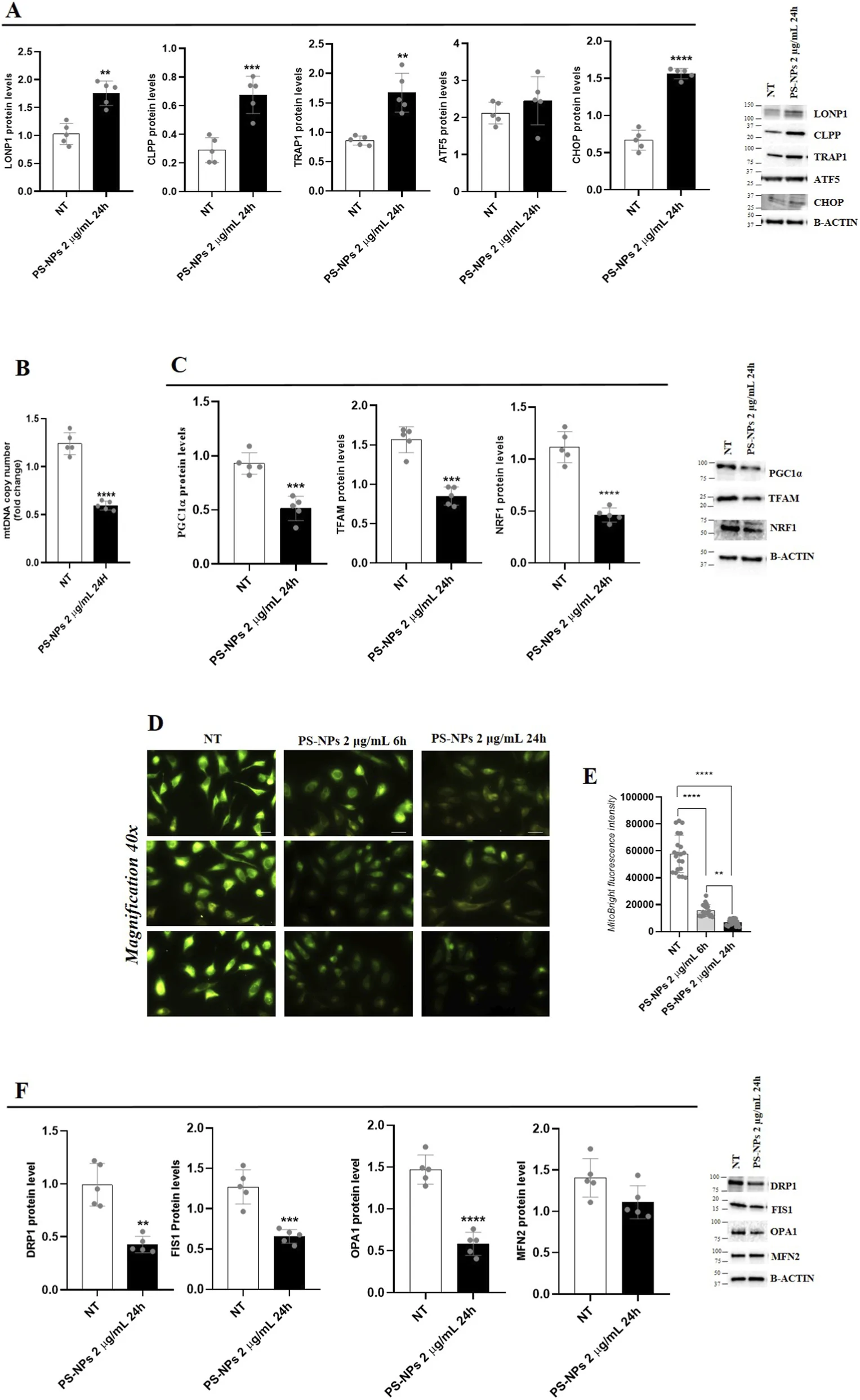
*PS-NPs alter mitochondrial quality control processes in HeLa cells.* **(A,C,F)** Representative immunoblots of LONP1, CLPP, TRAP1, ATF5, CHOP, PGC1α, TFAM, NRF1, DRP1, FIS1, OPA1 and MFN2, in HeLa cells after 24 h exposure to 2 μg/mL PS-NPs. Protein levels were normalized to β-ACTIN, and densitometric quantification is shown. The displayed blot is a representative cropped image; full-length uncropped blots are provided in the Supplementary Material. **(B)** mtDNA copy number was quantified by qPCR as the ratio between a mitochondrial gene (COXII) and a nuclear gene (β-ACTIN). **(D,E)** MitoBright Green staining; representative image of Mitobright Green Probe positive cells (green) acquired at ×40 (scale bar: 40 µm) and quantified using ImageJ. Data are shown as individual data points with mean ± SD from n = 5 independent biological replicates. Statistical analysis was determined using one-way ANOVA with Tukey’s *post hoc* test or Student’s t-test where appropriate. **p < 0.01, ***p < 0.001, ****p < 0.0001 vs. NT.

Western blot analysis revealed that protein levels of peroxisome proliferator-activated receptor gamma coactivator 1α (PGC1α), nuclear respiratory factor 1 (NRF1), and mitochondrial transcription factor A (TFAM), key markers of mitochondrial biogenesis, were significantly decreased in PS-NP-treated cells compared with control cells (Figure 4C). Consistent with these findings, MitoBright Green fluorescence was significantly reduced following PS-NP exposure at both 6 and 24 h compared with untreated cells (Figures 4D,E), indicating a decrease in mitochondria-associated fluorescence. To obtain a more comprehensive overview of mitochondrial biogenesis and abundance, we quantified mitochondrial DNA (mtDNA) copy number, which represents an indicator of mitochondrial genome abundance within cells (Abu et al., 2023). Our results showed that mtDNA copy number was significantly decreased in PS-NP-treated cells compared with controls, indicating a reduced number of mitochondrial genome copies per cell (Figure 4B). Taken together, the reductions in MitoBright Green fluorescence, mtDNA copy number, and PGC1α, NRF1, and TFAM protein levels are consistent with impaired mitochondrial biogenesis and reduced mitochondrial abundance following PS-NP exposure.

Next, we assessed the protein expression of key markers associated with mitochondrial fission and fusion following exposure to PS-NPs. Our results showed a significant decrease in the protein levels of Dynamin-Related Protein 1 (DRP1) and Mitochondrial fission one protein (FIS1), markers of mitochondrial fission, as well as Optic Atrophy 1 (OPA1), a marker of mitochondrial fusion, in PS-NP-treated cells compared to control cells (Figure 4F). In contrast, the protein levels of Mitofusin 2 (MFN2) showed a decrease of approximately 20% in PS-NP-treated cells compared to the control group (Figure 4F).

Finally, we investigated the effects of PS-NPs exposure on autophagy- and mitophagy-related processes. The protein levels of autophagy markers, including sequestosome 1 (SQSTM1/p62) and microtubule-associated protein one light chain three isoform B (LC3B), were measured. SQSTM1/p62 functions as an autophagosome cargo receptor, directing proteins that interact with it toward selective autophagy pathways. LC3B plays a central role in the formation and structure of the autophagosome membrane. During the autophagic process, p62 is degraded, while LC3B is upregulated; therefore, reduced levels of p62 are commonly associated with the activation of the autophagy pathway. Our results showed a significant increase in the LC3B-II/I ratio and a significant decrease in the protein levels of SQSTM1/p62 in PS-NP-treated cells compared to the control group (Figure 5B). The autophagy immunofluorescence assay showed that, 24 h after PS-NP exposure, the percentage of cells positive for the autophagosome detection reagent was increased compared to control conditions (Figure 5A). Furthermore, we observed in PS-NP-treated cells, compared to the control group, a significant decrease in the protein levels of Parkin RBR E3 ubiquitin ligase (PARKIN), AMBRA1, PINK1, all of which are markers of mitophagy, (Figure 5C). Thus, the activation of autophagy, coupled with a reduction in mitophagy, suggests an imbalance between these two processes, which may contribute to the accumulation of damaged mitochondria, thereby promoting oxidative stress, energetic dysfunction, and impaired MQC.

**FIGURE 5:**
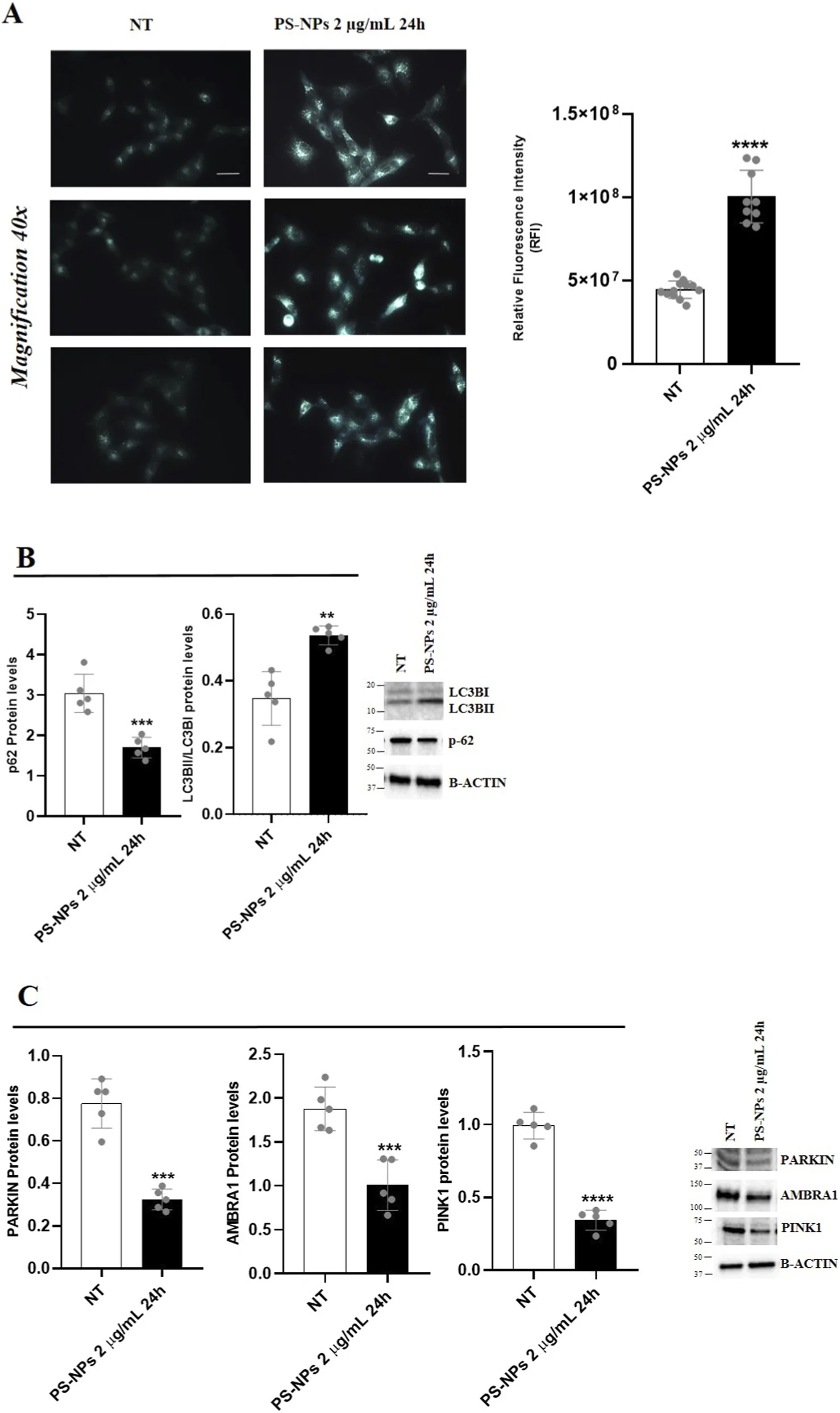
*PS-NP exposure differentially affects autophagy and mitophagy in HeLa cells*. **(A)** Autophagic cells detected in HeLa cells treated with 2 μg/mL PS-NPs for 24 h. Images were acquired at ×40 magnification (scale bars: 40 µm) and quantified using ImageJ. **(B,C)** Representative immunoblots of LC3B-I/II, p62, AMBRA1, PARKIN, and PINK1 in HeLa cells after 24 h exposure to 2 μg/mL PS-NPs. Protein levels were normalized to β-ACTIN, and densitometric quantification is shown. The displayed blot is a representative cropped image; full-length uncropped blots are provided in the Supplementary Material. Data are shown as individual data points with mean ± SD from n = 5 independent biological replicates. Statistical analysis was determined using one-way ANOVA with Tukey’s *post hoc* test or Student’s t-test where appropriate. **p < 0.01, ***p < 0.001, ****p < 0.0001 vs. NT.

### 3.4 PS-NP exposure induces ER stress and alters Mitochondria–ER interactions in HeLa cells

PS-NPs also induced ER stress, as evidenced by the upregulation of several ER stress markers. Specifically, our results showed that the protein levels of binding immunoglobulin protein (GRP78/BiP), a key indicator of ER stress, along with the phosphorylation levels of PKR-like endoplasmic reticulum kinase (PERK (Thr980)) and eukaryotic translation initiation factor 2 subunit alpha (eIF2α (Ser51)), were significantly increased in PS-NP-treated cells compared to the control group (Figure 6A). Additionally, the protein levels of ATF4 were significantly increased in HeLa cells exposed to PS-NPs compared to controls (Figure 6A). This suggests that PS-NPs exposure triggers a marked ER stress response in HeLa cells with the activation of UPR^ER^. Finally, we investigated the effects of PS-NPs on mitochondria–ER interactions in HeLa cells by analyzing the protein levels of Inositol 1,4,5-trisphosphate receptor (IP3R), glucose-regulated protein 75 (GRP75), and voltage-dependent anion channel 1 (VDAC1). The IP3R–GRP75–VDAC1 complex is a key mediator of communication between the endoplasmic reticulum and mitochondria. It facilitates efficient calcium transfer from the ER to mitochondria, a process crucial for mitochondrial metabolism, ATP production, and the regulation of cell survival and apoptosis (De Ridder et al., 2023). Western blot analysis revealed a significant upregulation of IP3R, GRP75, and VDAC1 protein levels in HeLa cells exposed to PS-NPs compared to the control group (Figure 6C). Subsequently, intracellular calcium levels were measured. Our results showed a significant increase in intracellular calcium in cells exposed to PS-NPs for 24 h compared to controls (Figure 6B). These findings highlighted that PS-NPs induce ER stress and disrupt ER–mitochondria communication, leading to altered calcium homeostasis.

**FIGURE 6:**
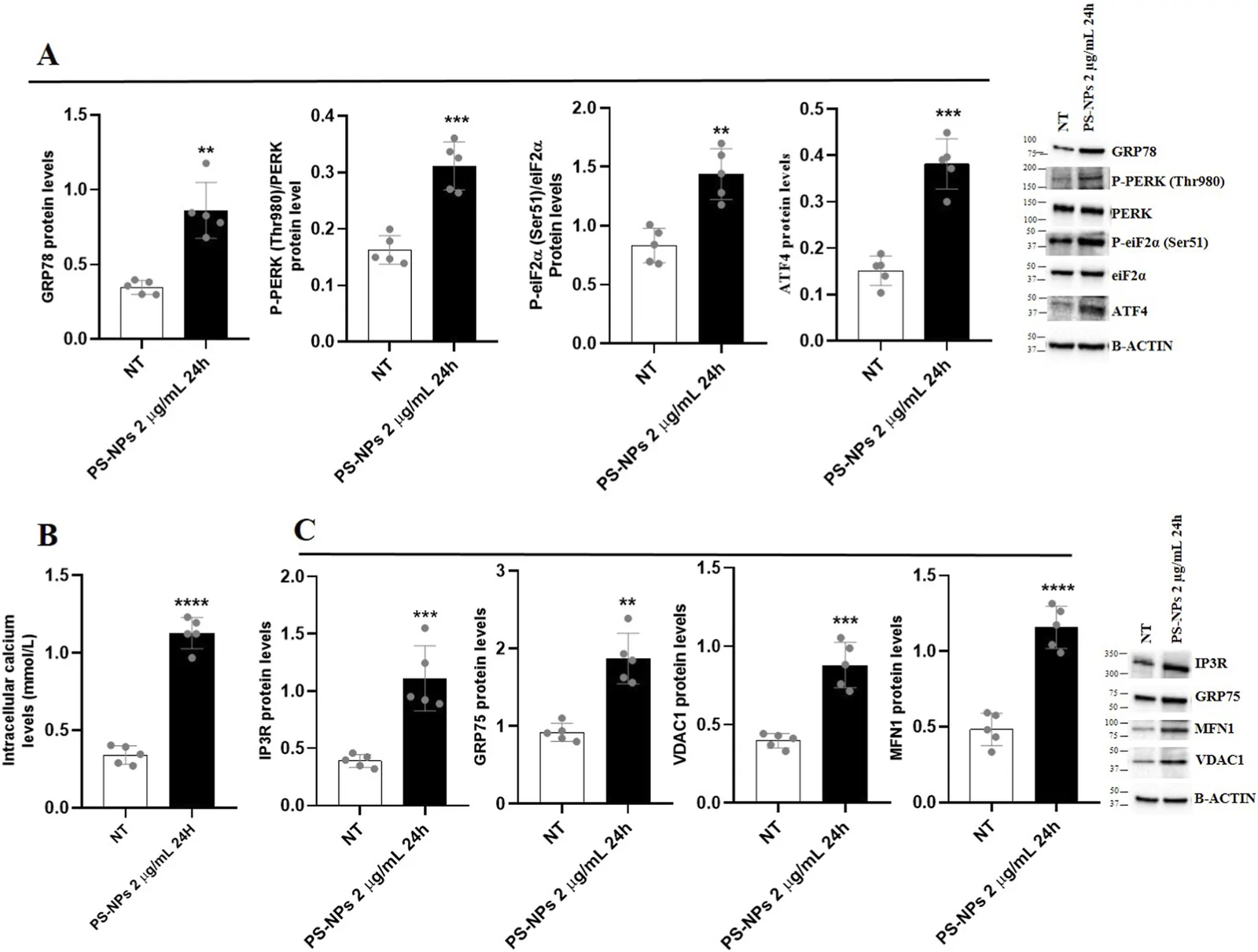
*PS-NPs induce ER stress and impair ER–mitochondria crosstalk in HeLa cells.* **(A,C)** Representative immunoblots of GRP78, P-PERK (Thr980), PERK, P-eIF2α (Ser51), eIF2α, IP3R, GRP75, MFN1, and VDAC1 in HeLa cells after 24 h exposure to 2 μg/mL PS-NPs. Protein levels were normalized to β-ACTIN and/or total protein for phosphorylated forms, and densitometric quantification is shown. The displayed blot is a representative cropped image; full-length uncropped blots are provided in the Supplementary Material. **(B)** Intracellular Ca^2+^ levels (reported as mmol/L) in cells treated with PS-NPs for 24 h. Data are shown as individual data points with mean ± SD from n = 5 independent biological replicates. Statistical analysis was determined using one-way ANOVA with Tukey’s *post hoc* test or Student’s t-test where appropriate. **p < 0.01, ***p < 0.001, ****p < 0.0001 vs. NT.

### 3.5 PS-NPs exposure induces apoptosis in HeLa cells

Sustained endoplasmic reticulum (ER) stress results in the accumulation of misfolded proteins, thereby triggering the activation of apoptotic signaling pathways (Ghemrawi and Khair, 2020). We measured the protein levels of Caspase-3, Bad, and Bax, which are key mediators of programmed cell death. We also assessed Bcl-2, an anti-apoptotic protein. Western blot analysis showed a significant upregulation of Caspase-3 protein levels, whereas Bcl-2 protein levels were markedly downregulated in PS-NP-treated cells compared to controls (Figure 7). As for Bad and Bax, their protein levels showed a reduction, although not statistically significant, in PS-NP-treated cells compared to controls (Figure 7). Together, these findings support the activation of apoptotic signaling following PS-NP exposure.

**FIGURE 7:**
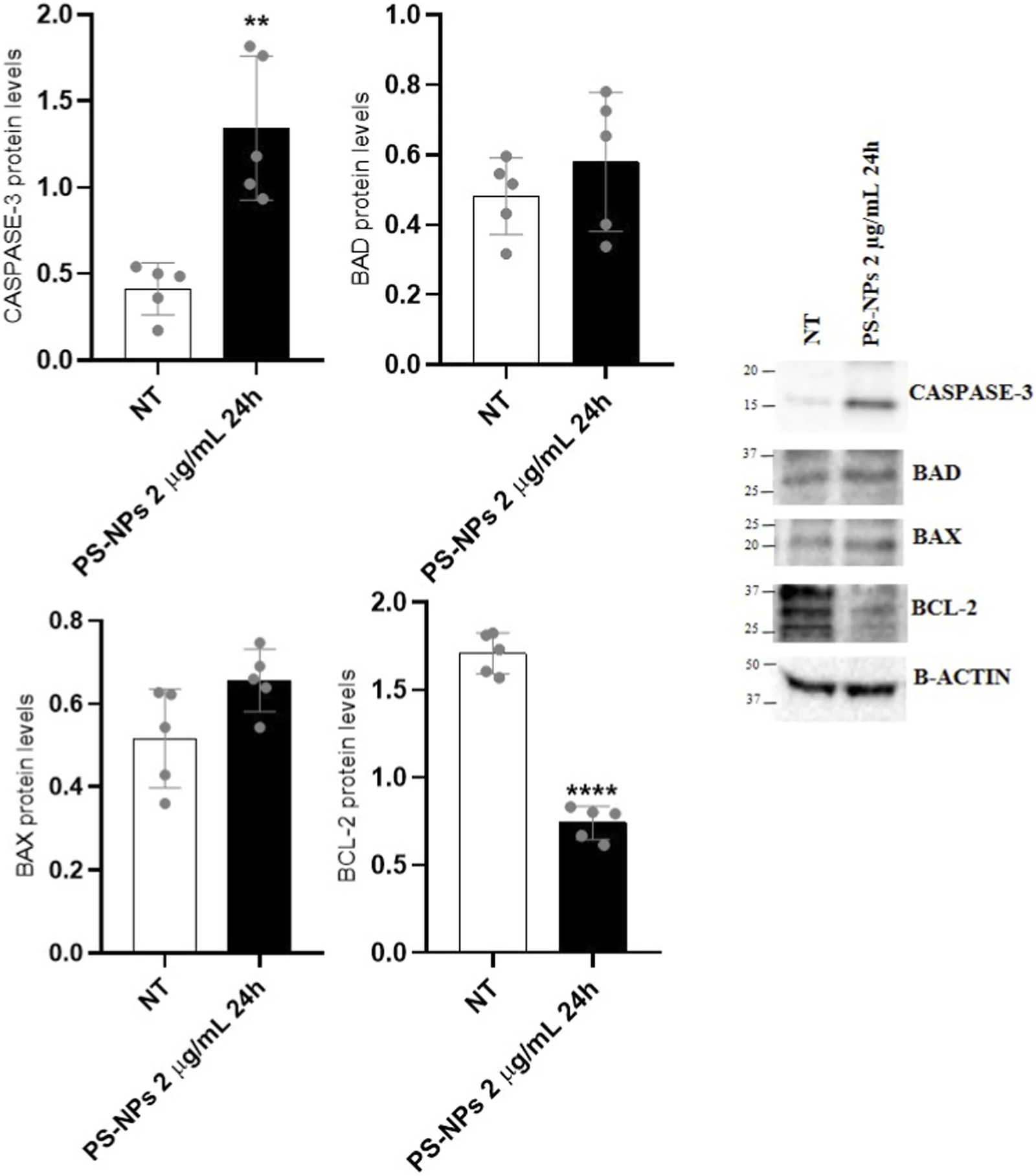
*PS-NPs trigger apoptotic signaling in HeLa cells.* Representative immunoblots of Caspase-3, BAX, BAD, and Bcl-2 in HeLa cells after 24 h exposure to 2 μg/mL PS-NPs. Protein levels were normalized to β-ACTIN, and densitometric quantification is shown. The displayed blot is a representative cropped image; full-length uncropped blots are provided in the Supplementary Material. Data are shown as individual data points with mean ± SD from n = 5 independent biological replicates. Statistical analysis was determined using one-way ANOVA with Tukey’s *post hoc* test or Student’s t-test where appropriate. **p < 0.01, ****p < 0.0001 vs. NT.

## 4 Discussion

Mitochondria are a primary target of NP-induced toxicity, as supported by multiple *in vivo* and *in vitro* studies showing mitochondrial damage mediated by oxidative stress, physical disruption, and impaired energy metabolism (Liu et al., 2025; Sun et al., 2023). These observations gain particular significance considering the increasing human exposure to nanoplastics, reinforcing concerns about the risks of environmental contamination. Given their persistence, environmental diffusion, and ability to accumulate in biological systems, nanoplastics are increasingly recognized as emerging environmental contaminants with potential implications for ecosystem and human health (Wang et al., 2021; Sana et al., 2020). In this context, the present study investigated the cellular mechanisms underlying the response to PS-NP exposure in HeLa cells, with particular emphasis on mitochondrial bioenergetics, mitochondrial quality control, and ER–mitochondria communication. The biological relevance of the experimental model is supported by our previous investigations demonstrating efficient internalization of the same PS-NP formulation in HeLa cells and the induction of intracellular molecular alterations, including disruption of protein homeostasis and DNA damage responses (Feola et al., 2025; Della Valle et al., 2022).

The electron transport chain plays a central role in aerobic respiration, and its dysfunction compromises oxidative phosphorylation, enhances ROS generation, and disrupts cellular energy homeostasis. In line with this, several reports indicate that NPs alter mitochondrial respiration and ATP production (Liu et al., 2025; Lin et al., 2022; Trevisan et al., 2019; Bonanomi et al., 2022).

We first investigated the effects of PS-NP exposure on mitochondrial bioenergetics. PS-NP-treated cells displayed increased basal and FCCP-stimulated respiration, indicating enhanced mitochondrial oxygen consumption under both basal and FCCP-stimulated conditions. The increase in basal OCR may represent a compensatory attempt to sustain cellular energy production in response to mitochondrial stress and potentially increased energetic demand. However, this enhanced oxygen consumption was not accompanied by a corresponding increase in ATP-linked respiration. Instead, proton-leak respiration was significantly increased following PS-NP exposure, while coupling efficiency was reduced, indicating that mitochondrial oxygen consumption became less efficiently coupled to ATP synthesis. A comparable respiratory profile, characterized by increased basal, oligomycin-insensitive and uncoupler-stimulated respiration, has been associated with increased mitochondrial leak and reduced coupling in other cellular models (Ozcan et al., 2026). Thus, although the increase in basal respiration may reflect an attempt to sustain cellular energy production, the increased proton leak renders this response bioenergetically inefficient.

Consistently, PS-NP-treated cells exhibited reduced cellular ATP levels and a significant loss of mitochondrial membrane potential, indicating mitochondrial depolarization and further supporting the occurrence of mitochondrial dysfunction, in agreement with previous reports (Lin et al., 2022; Sun et al., 2024; Halimu et al., 2022). Importantly, the ATP assay measured the total cellular ATP pool at the experimental endpoint and did not distinguish between ATP generated through oxidative phosphorylation and glycolysis. Therefore, the observed reduction in cellular ATP levels cannot be attributed specifically to impaired mitochondrial ATP production. The concomitant increase in extracellular L-lactate levels suggests an enhanced glycolytic contribution, potentially representing an additional compensatory response to mitochondrial stress and increased energetic demand. Nevertheless, cellular ATP levels remained reduced despite the increase in extracellular lactate, suggesting that the compensatory metabolic response was insufficient to maintain ATP homeostasis and that cellular ATP utilization was increased.

Consistent with this interpretation, a comparable metabolic profile, characterized by increased lactate levels together with reduced cellular ATP, has been reported following polystyrene nanoparticles exposure and was associated with a metabolic shift toward glycolysis (Peng et al., 2024; Bonanomi et al., 2022; Cheng et al., 2025). In addition, the restoration and maintenance of ionic and calcium gradients under conditions of mitochondrial stress may impose a substantial energetic demand, thereby further contributing to the reduction in cellular ATP levels. Taken together, our respirometric and metabolic findings indicate that PS-NP exposure elicits compensatory respiratory and glycolytic response that are nevertheless insufficient to preserve cellular energy homeostasis. The increased proton-leak respiration and reduced coupling efficiency specifically support impaired mitochondrial coupling rather than a generalized inhibition of the electron transport chain. This interpretation is consistent with previous studies reporting structural and functional mitochondrial alterations following PS-NP exposure across different cellular models (Fan et al., 2024; Domenech et al., 2021; Ragusa et al., 2025).

Mitochondrial dysfunction and cellular redox imbalance are closely interconnected processes (Chen et al., 2024; Suski et al., 2012). Concomitant with the observed reduction in MMP, PS-NP-treated HeLa cells exhibited significantly increased H_2_O_2_ levels in cell lysates at both exposure times. The increased H_2_O_2_ levels were accompanied by a marked reduction in key antioxidant enzymes, including SOD-2, GPX-1, and GPX-4, indicating impaired redox homeostasis. Although the present study does not allow us to establish the temporal sequence or causal relationship between these events, their concomitant occurrence, together with previous evidence of increased ROS levels following PS-NP exposure, suggests that sustained redox imbalance may contribute to the impairment of cellular antioxidant defense systems. Indeed, persistent ROS accumulation has been reported to disrupt antioxidant defenses through oxidative modifications of proteins and dysregulation of redox-sensitive signalling pathways, thereby reducing cellular antioxidant capacity and establishing a feed-forward cycle that may further amplify oxidative stress (Giannandrea et al., 2024; Adler et al., 2024; Shan et al., 2022; Sikder et al., 2025; He et al., 2017; Poljsak et al., 2013; Manful et al., 2025; Kik et al., 2021).

Given the extensive mitochondrial impairment, we decided to investigate whether PS-NP exposure affected MQC mechanisms. MQC encompasses interconnected processes such as UPR^mt^-associated responses, mitochondrial biogenesis, dynamics, and mitophagy, which collectively contribute to the maintenance of mitochondrial integrity and cellular homeostasis. Our data demonstrate that PS-NPs are associated with marked alterations in these adaptive systems. The UPR^mt^ represents an adaptive mitochondrial stress response involved in the maintenance of mitochondrial proteostasis under conditions of mitochondrial dysfunction (Shpilka and Haynes, 2018). Although UPR^mt^-associated responses have been reported in non-human models following NP exposure (Liu and Wang, 2021), our study provides, to the best of our knowledge, the first evidence that PS-NP exposure induces a mitochondrial stress response associated with the modulation of multiple UPR^mt^-related proteins in human cells. These findings expand the current understanding of nanoplastic-induced stress responses beyond oxidative damage and highlights mitochondrial proteostasis as a previously underexplored target of PS-NP toxicity. Specifically, PS-NP exposure significantly increased the levels of mitochondrial proteases LONP1 and CLPP, as well as the chaperone TRAP1 and the transcription factors ATF4, ATF5, and CHOP. While transient engagement of UPR^mt^-associated mechanisms may support mitochondrial recovery, persistent mitochondrial stress and prolonged activation of stress responses may contribute to mitochondrial dysfunction and cellular damage (Chen et al., 2025).

In parallel, PS-NP exposure markedly impaired mitochondrial biogenesis, as indicated by reduced levels of PGC1α, NRF1, and TFAM, along with a decrease in mitochondrial DNA copy number. This interpretation was further supported by the significant reduction in MitoBright Green fluorescence observed in PS-NP-treated cells. Moreover, we observed a disruption of mitochondrial dynamics, characterized by reduced expression of fission markers FIS1 and DRP1 and the fusion protein OPA1. These alterations likely contribute to the accumulation of dysfunctional mitochondria, in agreement with previous reports on MPs/NPs-induced dysregulation of mitochondrial morphology and function (Li et al., 2023; Fu et al., 2022; Im et al., 2022; Liu et al., 2022; Zhang et al., 2022).

Autophagy and mitophagy are essential for the selective removal of damaged mitochondria. While NP exposure has been reported to activate autophagy as an adaptive response (Fanghella et al., 2025), our data reveal a more complex condition. PS-NP-treated cells displayed increased LC3BII/LC3BI ratio and reduced p62 levels, along with enhanced autophagic signal by immunofluorescence. However, this activation was accompanied by a significant reduction in PINK1, PARKIN, and AMBRA1 protein levels, indicating impaired mitophagy. These findings suggest that PS-NPs exposure trigger autophagosome accumulation without effective mitochondrial clearance, consistent with impaired mitophagy and/or altered autophagic flux (Fanghella et al., 2025; Wong and Cuervo, 2010). Notably, suppression of PINK1/PARKIN-mediated mitophagy under oxidative stress conditions, concomitant with UPR^mt^ modulation, has been previously reported (Wang et al., 2023).

Oxidative stress also promotes ER proteotoxic stress and activation of the UPR^ER^ (Ong and Logue, 2023). In PS-NP-treated HeLa cells, we observed robust induction of ER stress markers, including GRP78/BiP, phosphorylated PERK and eIF2α, and increased ATF4 protein levels, indicating activation of the PERK branch of the UPR^ER^. Prolonged UPR^ER^ activation may shift from adaptive signaling to apoptosis (Wang and Kaufman, 2012). The ER and mitochondria are functionally linked via MAMs, which play a key role in regulating calcium homeostasis, mitochondrial dynamics, and cellular fate decisions (Gao et al., 2020). The IP3R–GRP75–VDAC complex is a hallmark of MAMs (Zhang et al., 2023; Greene et al., 2025).

Our results demonstrate that PS-NP exposure significantly increased the protein levels of IP3R, GRP75, and VDAC, indicating altered expression of MAM-associated proteins, consistent with changes in ER–mitochondria contact regulation. This was accompanied by a pronounced increase in intracellular calcium levels. These findings are consistent with previous observations showing that NPs promote MAM assembly and excessive ER-to-mitochondria calcium transfer, leading to mitochondrial calcium overload (Wei et al., 2024). Moreover, these changes are consistent with conditions that may lead to mitochondrial calcium overload, destabilizing mitochondrial membranes, promotes cytochrome c release, and activates apoptotic pathways (Simmen et al., 2005; Giorgi et al., 2012). Accordingly, PS-NP-treated cells exhibited increased Caspase-3 levels and a concomitant reduction in the anti-apoptotic protein Bcl-2, supporting the involvement of mitochondria-dependent apoptosis. Disruption of ER–mitochondria signaling may therefore represent an important mechanism by which PS-NP exposure contributes to cellular dysfunction.

Overall, our study provides novel mechanistic insights into PS-NP-induced toxicity, identifying mitochondria as central targets. Our findings indicate that PS-NP exposure is associated with impaired mitochondrial coupling, oxidative stress, disruption of MQC pathways, altered ER–mitochondria communication and apoptotic cell death. These findings significantly advance our understanding of nanoplastic-induced cellular stress and underscore the critical role of mitochondrial dysfunction in mediating PS-NP toxicity.

A key strength of this work lies in the identification, in a human cell model, of a mitochondrial stress response involving the modulation of multiple UPR^mt^-associated proteins following PS-NP exposure. The concomitant modulation of UPR^mt^ signaling, suppression of mitophagy, and enhanced MAM-dependent calcium transfer suggests that nanoplastics do not simply damage mitochondria, but may contribute to a coordinated mitochondrial stress response involving UPR^mt^ signaling, altered mitophagy, and changes in ER–mitochondria communication. Importantly, our data indicate that these adaptive pathways may become maladaptive upon sustained activation, ultimately contributing to mitochondrial failure and cell death.

Despite these advances, several limitations of the present study should be acknowledged. First, our analyses were performed in a single transformed human cell line, which, while widely used for mechanistic studies, may not fully recapitulate the responses of primary or tissue-specific cells. However, the primary objective of the present study was not to investigate tissue-specific responses to PS-NPs, but rather to elucidate the cellular mechanisms underlying nanoplastic-induced toxicity, with particular emphasis on mitochondrial bioenergetics, MQC, and ER–mitochondria communication. These pathways are highly conserved across mammalian cell types and represent fundamental components of the cellular stress response. In addition, HeLa cells provide a robust and widely validated platform to investigate these conserved pathways and responses induced by nanoplastic exposure and have been extensively used as a reference model for mechanistic studies of nanoparticle uptake, intracellular trafficking, and nanoparticle-induced cellular responses, thereby enabling direct comparison with a large body of existing literature (Åberg et al., 2021; Sandin et al., 2012; Jiang et al., 2010; Schöttler et al., 2016; Panarella et al., 2016). Second, the use of commercially available monodisperse PS-NPs may not fully reproduce the physicochemical heterogeneity of environmentally derived nanoplastics. Since the present study was based on acute *in vitro* exposure (6 and 24 h), which does not fully reproduce the cumulative effects and adaptive cellular responses associated with chronic environmental exposure to nanoplastics, future studies employing long-term exposure protocols will be essential to validate the mechanisms identified here under more environmentally relevant exposure conditions. These limitations open important avenues for future research. Extending these findings to primary cells and organ-specific models will be essential to evaluate the biological and physiological relevance of PS-NP-associated mitochondrial alterations.

## 5 Conclusion

This study underscores the vulnerability of mitochondrial homeostasis to nanoplastic exposure and positions MQC disruption and ER–mitochondria crosstalk at the core of PS-NP–induced cellular toxicity. Overall, these findings suggest that disruption of MQC may represent a common mechanism underlying nanoplastic toxicity, providing a mechanistic framework for future studies aimed at elucidating the toxicological and pathophysiological consequences of environmental nanoplastic exposure.

## Data Availability

The raw data supporting the conclusions of this article will be made available by the authors, without undue reservation.
