# Supplementary material for: Polystyrene nanoplastics impair mitochondrial function, quality control, and ER–mitochondria crosstalk in HeLa cells
Source: Front Toxicol. 2026 Oct 5;8:1907794. doi: 10.3389/ftox.2026.1907794 (PMC13677859; doi:10.3389/ftox.2026.1907794)
Supplement: Supplementary file 1 [file DataSheet2.pdf]

Supplementary Material

1 Supplementary Text

S1. Evaluation of Oxygen Consumption Rate (OCR) of intact cells

Oxygraph-2K respirometer (Oroboros, Innsbruck, Austria) was utilized to assess oxygen consumption. After trypsin detachment, cells were resuspended in RPMI-1640 media and then centrifuged at 350×g for 5 minutes at room temperature. The pellet was resuspended in RPMI-1640 to achieve 1×10^6 cells/mL. Following instrument calibration 2 mL of the prepared cell suspension was added. The OCR was evaluated under controlled conditions. Multiple respiratory states were assessed: Routine respiration, which occurred in the absence of added substrates; Oligomycin-sensitive respiration stimulated by Oligomycin (Merck, Darmstadt, Germany) (2.5 µM) which inhibited ATP synthase; and Maximum respiration was assessed by the addition of carbonyl cyanide-4-(trifluoromethoxy)phenylhydrazone (FCCP) (Merck, Darmstadt, Germany) (1 µM), a mitochondrial uncoupler. Finally, to inhibit mitochondrial respiration and assess non-mitochondrial oxygen consumption, Rotenone (Merck, Darmstadt, Germany) (0.5 µM) and Antimycin A (Merck, Darmstadt, Germany) (2.5 µM) were added to block complexes I and III, respectively.

S2. Assessment of ATP intracellular levels

Intracellular ATP concentrations were quantified using the Adenosine 5′-triphosphate (ATP) Assay Kit (Abbexa, Cambridge Science Park, UK) following the manufacturer’s protocol. In brief, the provided Extraction Solution was used for cell lysis, homogenization, and heating in a boiling water bath for 2 minutes. The samples were allowed to equilibrate to room temperature, after which they were centrifuged at 10.000 × g for 10 minutes. The resulting supernatant was then collected. ATP levels were measured by recording absorbance at 636 nm with a Synergy H1 microplate reader (BioTek, Shoreline, WA, USA) and subsequently normalized to the total protein content.

S3. Measurement of Intracellular Hydrogen Peroxide Levels

Hydrogen peroxide (H2O2) levels were evaluated using a commercial assay kit (Abcam, ab102500). A total of 2×10^6 cells were suspended in Assay Buffer and pelleted by centrifugation at 14,000 × g for 2–5 minutes at 4 °C. Proteins were removed by treating the samples with 4M perchloric acid (PCA),

followed by neutralization with ice-cold 2 M KOH, adjusting the pH to a final range of 6.5–8. The neutralized samples were then centrifuged at 13.000 × g for 15 minutes at 4 °C. Following the kit instructions, each well of a 96-well plate was dispensed with 50 µL of the reagent mix and 50 µL of the corresponding sample. After a short incubation, fluorescence intensity was recorded at 535 nm (excitation) and 587 nm (emission) using a Synergy H1 microplate reader (BioTek, Agilent, Santa Clara, CA, USA).

S4. Genomic DNA Isolation and mtDNA Copy Number by Real-Time PCR

Genomic DNA was extracted from HeLa cells using the Genomic-tip 20/G kit (Qiagen, Valencia, CA, USA), according to instruction by the kit. The concentration of the isolated DNA was determined using a Nanodrop One (Thermo Fisher Scientific, Waltham, MA, USA). Real-time quantitative PCR (qRT-PCR) was used to assess mtDNA copy numbers using StepOnePlusTM Real-Time PCR System (Thermo Fisher Scientific, Waltham, Massachusetts, USA). We analyzed the amplification of mitochondrial Cytochrome c oxidase subunit II (COII, mitochondrial-encoded gene) and β-actin (nuclear-encoded gene). In our study, the average Ct values of nuclear DNA and mtDNA were obtained for each case. mtDNA content was calculated using ΔCt = average Ct nuclear DNA - average Ct mtDNA and then by applying the formula mtDNA content = 2(2ΔCt). The list of primers used is reported in Table S2.

2. Supplementary Figures and Tables

2.1 Supplementary Table

Table S1. Antibodies used in this study for Western blot

Antibody	Reference number
AMBRA1	Cell Signaling -cod.#24907
ATF4	Abclonal -cod.#A18687
ATF5	AbClonal -cod.#A3563
B-ACTIN	Bioss Antibodies -cod.bs-0061R
BAD	Santa Cruz Biotechnology -cod.sc-8044
BAX	Santa Cruz Biotechnology -cod.sc-7480
BCL-2	Santa Cruz Biotechnology -cod.sc-7382
CASPASE-3	AbClonal -cod.#A2156
CATALASE	Sigma-Aldrich -cod.#C0979
CHOP	AbClonal -cod.#A0221
CLPP	AbClonal -cod.#A32214
DRP1	AbClonal -cod.#A2586
eiF2α (Ser51)	AbClonal -cod.#A0764
FIS1	AbClonal -cod.#A21527
GPX-1	GeneTex -cod. GTX03346
GPX-4	Abcam -cod.#ab125066
GRP75	AbClonal -cod.#A0558
GRP78	AbClonal -cod.#A0241

IP3R	AbClonal -cod.#A4436
LC3B	Santa Cruz Biotechnology -cod.sc-376404
LONP1	AbClonal -cod.#A4293
MFN1	AbClonal -cod.#A9880
MFN2	AbClonal -cod.#A19678
NRF1	AbClonal -cod.#A5547
OPA1	AbClonal -cod.#A9833
p62	Cell Signaling -cod.#5114
P-eiF2α (Ser51)	AbClonal -cod.#AP0745
P-PERK (Thr980)	Cell Signaling -cod.#3192
PARKIN	Cell Signaling -cod.#4211
PERK	AbClonal -cod.#AP1501
PGC1-α	Millipore -cod.#AB3242
PINK1	Abcam -cod.#ab186303
SOD-2	Santa Cruz Biotechnology -cod.sc-137254
TFAM	AbClonal -cod.#A3173
TRAP1	AbClonal -cod.#A2748
VDAC1	GeneTex –cod. GTX114187
Anti-rabbit IgG	Abcam –cod.#ab97051
Anti-mouse IgG	Abcam –cod.#ab97023

Table S2. List of primers

Gene name	Forward primer (5′→3′)	Reverse primer (5′→3′)
**COII**	TGAGCCATCCCTTCACTAGG	TGAGCCGCAAATTTCAGAG
**β-actin**	CTGCTCTTTCCCAGATGAGG	CCACAGCACTGTAGGGGTTT

2.2 Supplementary Figures

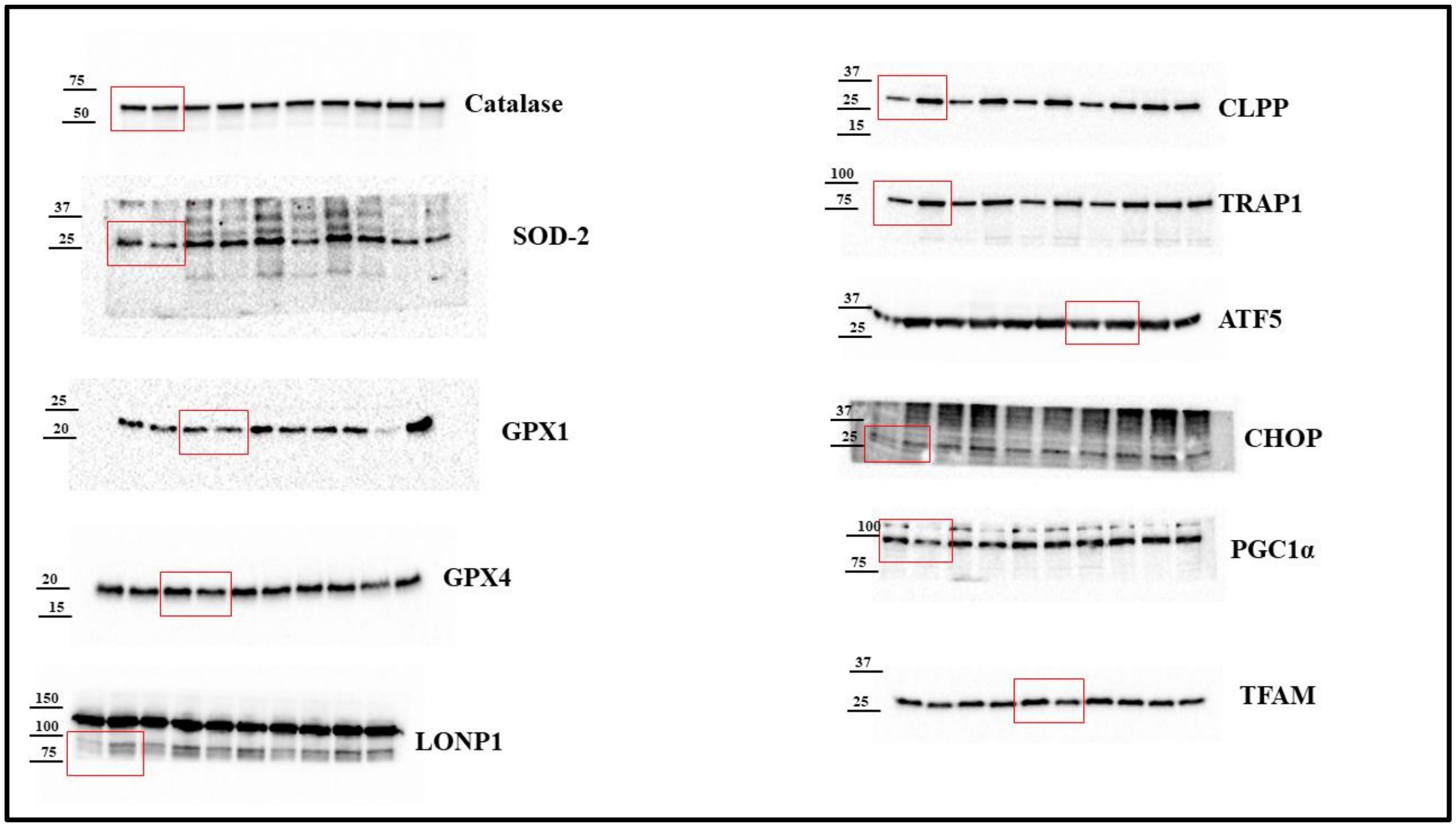

Supplementary Figure 1. Uncropped Western blot images

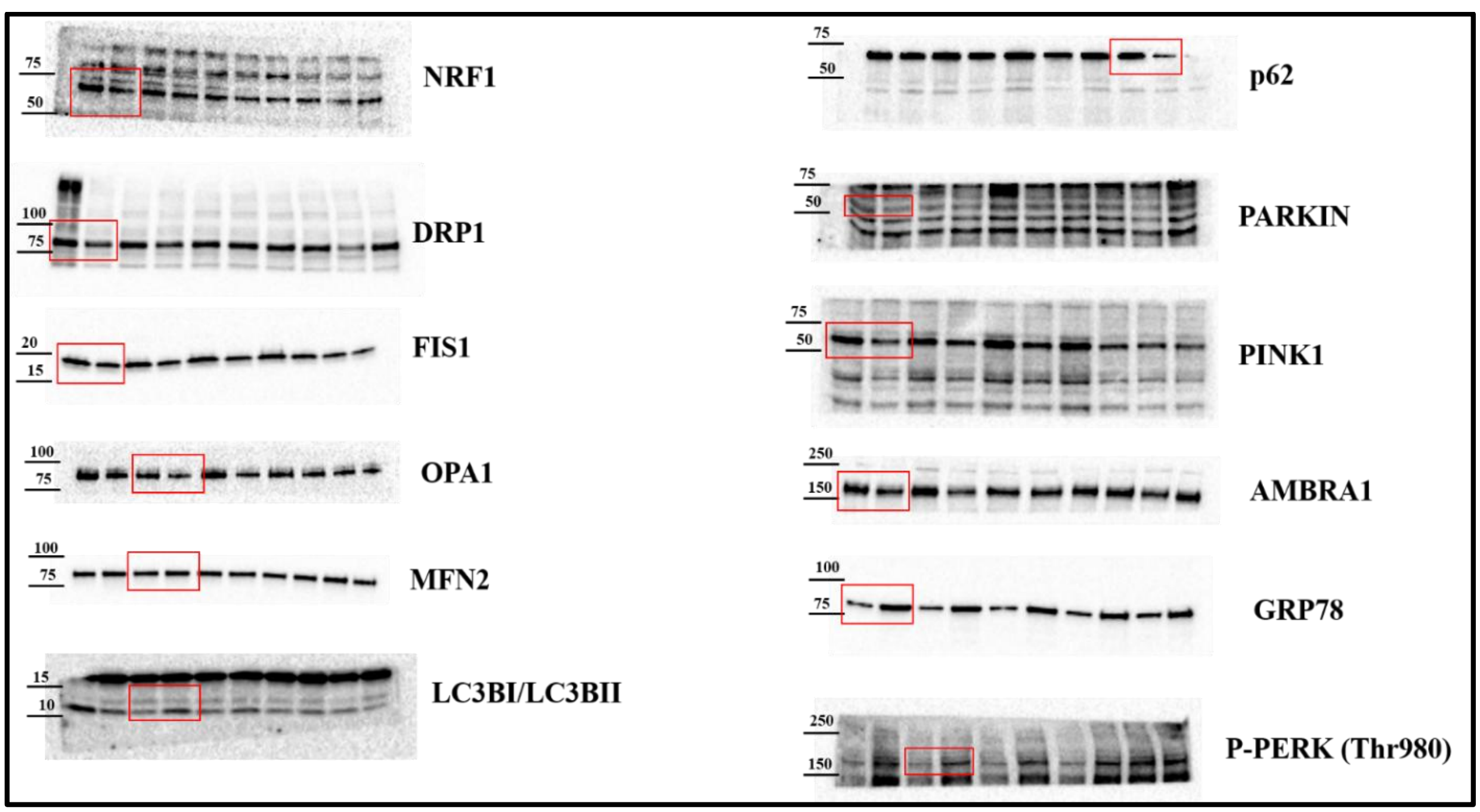

Supplementary Figure 2. Uncropped Western blot images

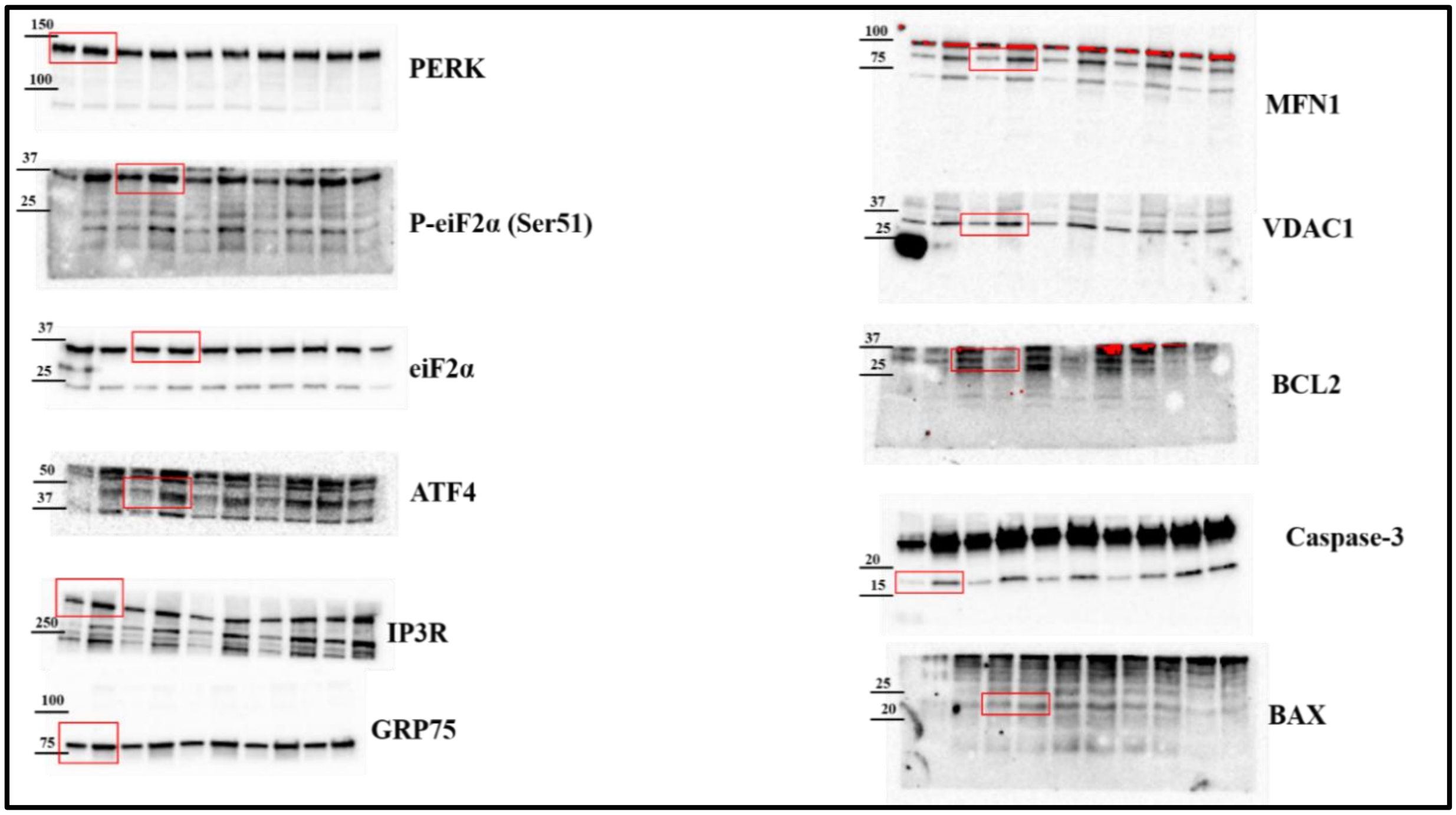

Supplementary Figure 3. Uncropped Western blot images

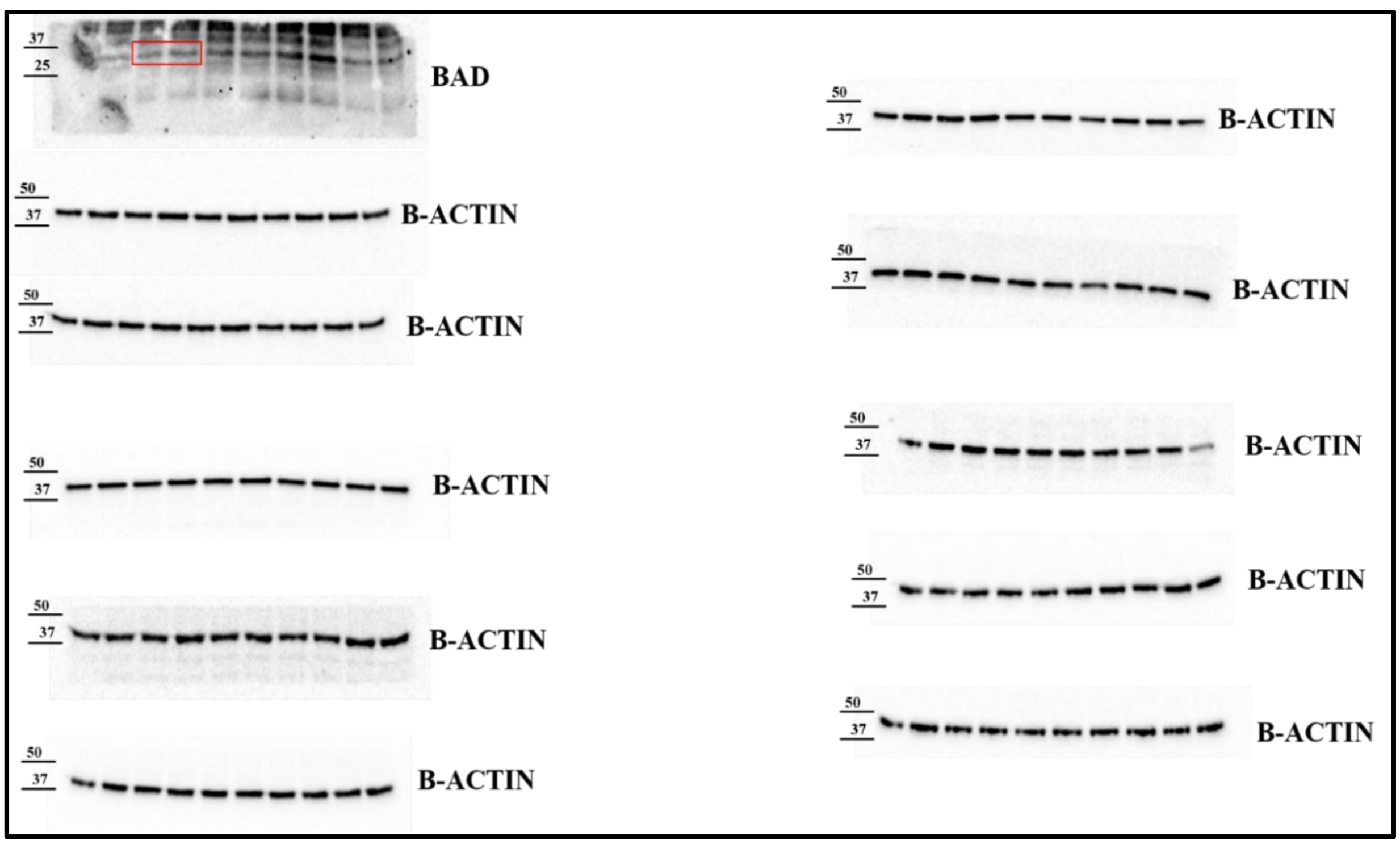

Supplementary Figure 4. Uncropped Western blot images

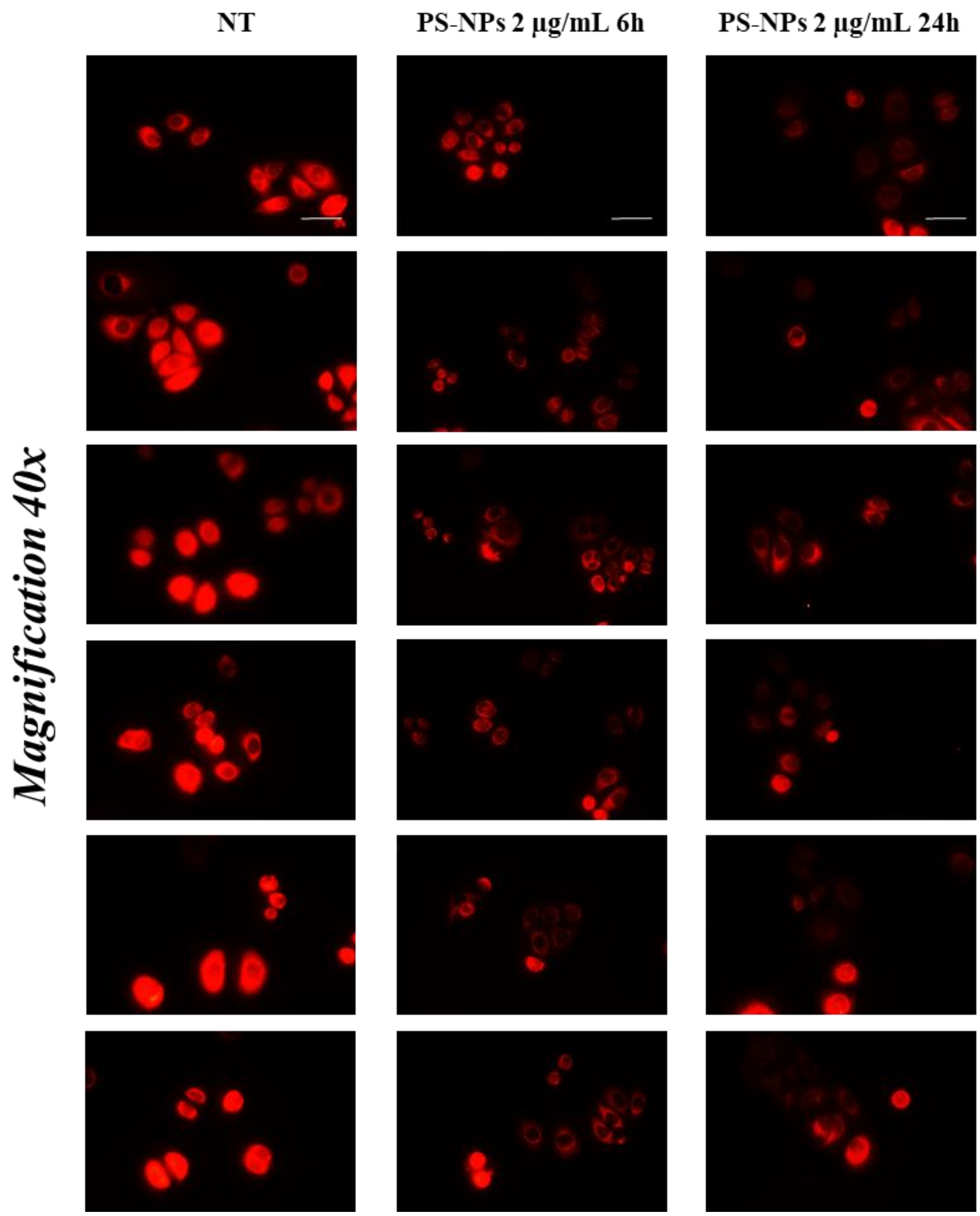

Supplementary Figure 5. Mitochondrial membrane potential assessed by TMRE staining, images of TMRE-positive cells (red) acquired at ×40 (scale bar: 40 µm).

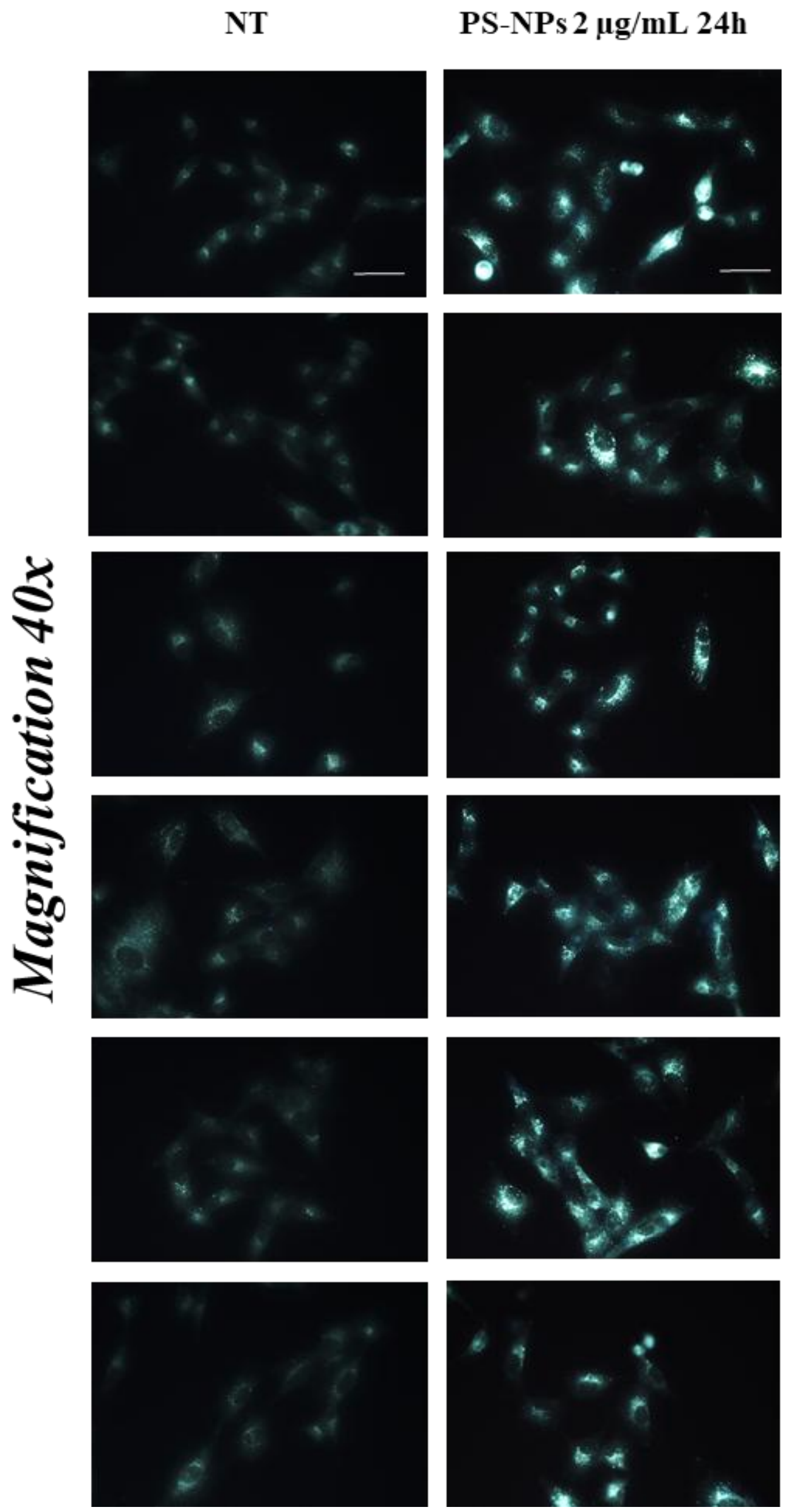

Supplementary Figure 6. Autophagic cells detected in HeLa cells treated with 2 µg/mL PS-NPs for 24 h. Images were acquired at ×40 magnification (scale bars: 40 µm).

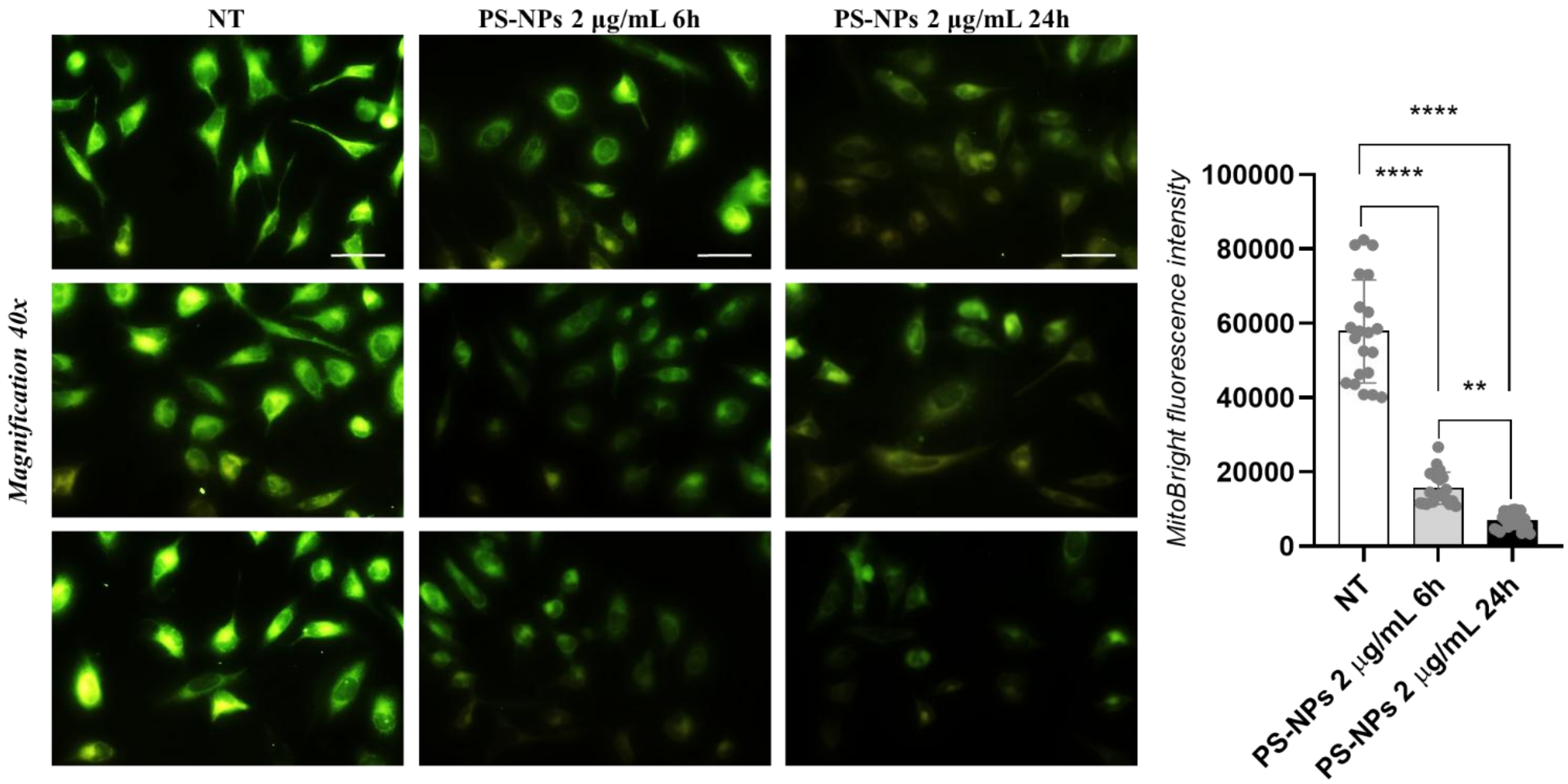

Supplementary Figure 7. Mitobright Green Probe positive cells treated with 2 µg/mL PS-NPs for 24 h. Images were acquired at ×40 magnification (scale bars: 40 µm).
